# Patient‐Reported Outcome Measures Used to Assess Surgical Interventions for Pelvic Organ Prolapse, Stress Urinary Incontinence and Mesh Complications: A Scoping Review for the Development of the APPRAISE PROM

**DOI:** 10.1111/1471-0528.18355

**Published:** 2025-09-24

**Authors:** Georgina Forshall, Kirsty Budds, Victoria Fisher, Swati Jha, Thomas G. Gray, Stergios K. Doumouchtsis, Anne‐Marie Bagnall, Georgina L. Jones

**Affiliations:** ^1^ Psychology, School of Humanities and Social Sciences Leeds Beckett University Leeds UK; ^2^ Department of Urogynaecology, Jessop Wing Sheffield Teaching Hospitals Sheffield UK; ^3^ Department of Urogynaecology and Pelvic Reconstructive Surgery Norfolk and Norwich University Hospitals NHS Foundation Trust Norwich UK; ^4^ Department of Obstetrics and Gynaecology Epsom and St. Helier University Hospitals NHS Trust Surrey UK; ^5^ St. George's University of London London UK; ^6^ Laboratory of Experimental Surgery and Surgical Research “N.S. Christeas” National and Kapodistrian University of Athens, Medical School Athens Greece; ^7^ School of Medicine American University of the Caribbean Cupecoy Sint Maarten; ^8^ School of Medicine Ross University Miramar Florida USA; ^9^ Centre for Health Promotion Research Leeds Beckett University Leeds UK

**Keywords:** mesh complications, NIHR, pelvic floor, pelvic organ prolapse, PREMs, PROMs, quality of life measurement, stress urinary incontinence, surgery

## Abstract

**Background:**

This scoping review was undertaken as part of an NIHR‐commissioned study, APPRAISE, to develop a patient‐reported outcome measure (PROM) and experience measure (PREM) to assess outcomes relevant to surgery for pelvic organ prolapse (POP), stress urinary incontinence (SUI) and pelvic mesh complications surgery, with cross‐cultural applicability.

**Objectives:**

To identify PROMs and PREMs used to assess POP, SUI and mesh complication surgery; to compare the length, recall periods, response options of these tools and the outcomes/experiences assessed.

**Search Strategy:**

Three databases searched from inception to September 2023 were screened by two independent reviewers.

**Selection Criteria:**

Primary studies using subjective measures to assess POP, SUI and mesh complication surgery for women aged 16+ years were eligible for inclusion. Related systematic reviews were also reviewed.

**Data Collection and Analysis:**

Data were extracted into a piloted electronic form by one reviewer and checked by a second. A narrative synthesis of the data was performed.

**Main Results:**

Of the 2079 included primary studies, 1607 (77%) used a PROM with evidence of psychometric testing. Five hundred and twenty‐two (25%) studies used one PROM; 1082 studies (52%) used two or more PROMs. One hundred and fifty‐one measures were extracted; of these, condition‐specific measures were the most highly cited. There was limited use of PROMs specific to surgery, mental health, body image and PREMs. Some outcomes (e.g., urinary symptoms, emotional wellbeing) are measured in a significantly higher proportion of PROMs than other outcomes.

**Conclusions:**

Currently, no existing validated PROM evaluates all patient‐reported outcomes relevant to surgery for POP, SUI or mesh complications.

## 1 Introduction

Pelvic floor disorders (PFDs), including stress urinary incontinence (SUI) and pelvic organ prolapse (POP), are multidimensional conditions with serious and often stigmatising consequences for patients' personal and social lives. A substantial number of patient‐reported outcome/experience measures (PROMs and PREMs) have been developed globally for use in urogynaecological clinical practice and research. Used alongside conventional history taking and clinical examination and investigations, PROMs are usually questionnaires evaluating patients' self‐reported perspectives of their health‐related quality of life (HRQL) or functionality [1, 2]. These measures provide rich data that cannot be obtained from conventional consultations alone [3], and assess a range of subjective outcomes from urinary, bowel and vaginal symptoms to sexual function, pain and discomfort, emotional wellbeing and related impacts on HRQL [4, 5]. PREMs, meanwhile, assess how patients perceive their experience of care, including satisfaction with treatment, information provision and involvement in decision making.

PROMs/PREMs play an important role in facilitating disclosure and discussion of intimate pelvic floor symptoms [4, 6] and they are also valuable instruments for research [7, 8, 9]. However, while these measures are well established in clinical practice, a consistent standardised approach to patient evaluation and outcome characterisation is lacking in the UK [5, 10, 11, 12, 13, 14, 15, 16]. In addition, limits to questionnaire content and scope have been identified and evidence of robust psychometric testing, including for widely used PROMs, is variable [4].

Concerns about serious complications associated with polypropylene mesh implants, used until recently in surgery for POP and SUI, were raised in the Cumberledge Review, *First Do No Harm*, which emphasised the need to hear from patients about the effect of surgery on their health and quality of life [17]. Consequently, the UK's National Institute for Health and Care Excellence (NICE) has called for the development and validation of universally accepted outcome and experience measures specific to surgery for POP, SUI and mesh complications [18].

This scoping review was undertaken as part of the NIHR‐commissioned project *A Patient‐reported outcome measure for PRolApse, Incontinence and meSh complication surgery (APPRAISE)* at Leeds Beckett University [19], to develop a comprehensive PROM and PREM for pelvic floor surgery, with cross‐cultural applicability for use in the UK. In accordance with best practice guidelines, the purpose of this scoping review was to inform the instruments' theoretical basis and content, including their conceptual framework, items and response formats. As such, the scope of this review was to identify *all* existing PROMs/PREMs, applied in primary studies of any design, that assess outcomes in female patients undergoing surgery for POP, SUI or pelvic mesh complications.

## 2 Methods

### 2.1 Review Questions

1. What instruments have been used to measure subjective surgical outcomes for POP, SUI and mesh complications in primary studies? How many of these have evidence of psychometric testing (i.e., are PROMs/PREMs with evidence of reliability, validity and responsiveness)?
2. What outcomes are assessed by these instruments?
3. What number of core items, recall periods and response options are used in these instruments?

The review protocol was registered on PROSPERO (CRD42023412745) [20] as a systematic review. However, following the pilot searches, given the scale and breadth of the included studies, a scoping review methodology was applied [21]. This was conducted in accordance with the Preferred Reporting Items for Systematic Reviews and Meta‐Analyses (PRISMA) guidelines [22]. A PRISMA flow diagram is provided in the results to illustrate the study selection process; a completed PRISMA checklist [23] is included in Appendix S1.

### 2.2 Search Strategy

Electronic databases MEDLINE, PsycINFO and CINAHL were searched from inception to September 2023 using search terms derived from existing reviews, which were also ‘mined’ for primary studies [4, 12, 13, 14, 15, 16, 24, 25, 26, 27, 28, 29, 30, 31]. A detailed search strategy is provided in Appendix S2.

### 2.3 Eligibility Criteria and Study Selection

Two reviewers (G.F., K.B., V.F. or A.M.B.) independently screened each title and abstract, and a single reviewer screened full text articles against the eligibility criteria using a piloted form in Covidence. Disagreements were resolved by consensus with reference to a third reviewer (G.F., K.B., V.F. or A.M.B.) or the study Advisory Group (G.J., T.G. and S.D.) as needed. The eligibility criteria were:

#### 2.3.1 Population

Women aged 16 years or over who have undergone surgical intervention for POP, SUI or for mesh complications (arising from surgical treatment for POP and SUI). Patients undergoing surgery for mixed urinary incontinence (MUI), unless specified stress‐dominant or hysterectomy for unspecified cause or a cause not related to POP (e.g., cancer) were excluded.

#### 2.3.2 Interventions

Subjective measures (e.g., existing PROMs/PREMs) used to surgical outcomes for POP, SUI and mesh complication surgery.

#### 2.3.3 Outcomes

Outcome domains, number of items, recall periods and response options of PROMs and PREMs used to measure surgical outcomes for POP, SUI and mesh complications.

#### 2.3.4 Types of Studies

Primary studies, both experimental and observational designs that used PROMs or PREMs to collect data on surgical outcomes in the populations above, including clinical audit studies. We excluded single patient case studies, case series of *n* < 5 [32] commentaries. Studies not reported in English were also excluded.

### 2.4 Data Extraction

Data from primary studies were extracted into the following categories onto a piloted electronic form, by one reviewer and checked by a second reviewer: publication author(s), date and study reference, the indication for surgery and type of subjective measure applied. Information relating to the identified PROMs/PREMs was recorded in an additional electronic form, including the name and purpose of the instrument, the related condition (if applicable), outcomes assessed, numbers of core and bother items, recall periods and response options.

### 2.5 Synthesis of Results

The results are presented in a narrative synthesis [33, 34], which first describes the study selection process and included studies, then presents a ‘map’ of the PROMs/PREMs used in primary studies, categorising the studies, extracted instruments and outcomes assessed, as well as describing the number of core items, recall periods and response options.

### 2.6 Stakeholder Engagement

Patient and public involvement (PPI) has been integral to the broader aims and development of the PROM and PREM produced by the APPRAISE project [19] and our patient user group has been closely involved in the development of the project's protocol, including the three systematic reviews being undertaken.

## 3 Results

**FIGURE 1:**
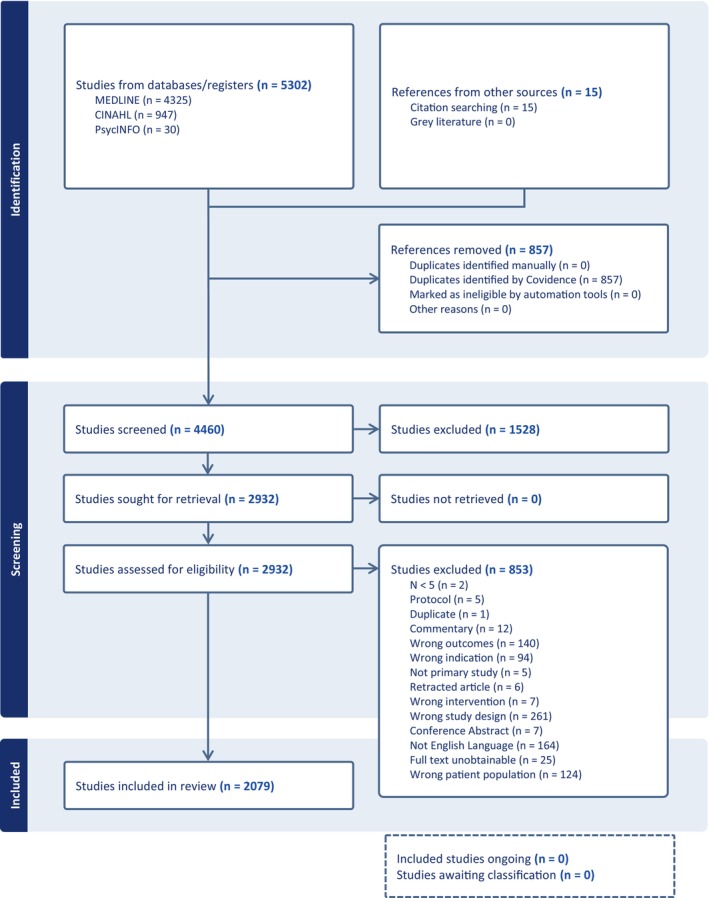
PRISMA flow chart.

A total of 5302 citations were retrieved from the database searches, as well as a further 15 from citation searching. Figure 1 provides the results of the search strategy within the PRISMA flow diagram. After the removal of duplicates, 4460 studies were identified for title and abstract screening. Papers were excluded during this stage of screening (*n* = 1528) and following the review of full texts (*n* = 853) if they did not meet the eligibility criteria. Included in the final review were 2079 primary studies that measured patient‐reported outcomes (PRO) in relation to surgery for POP, SUI or mesh complications, using either a PROM, PREM or an unvalidated or unspecified subjective measure (Table S1).

### 3.1 Categorisation of Studies

Included studies were categorised by the indication for and nature of surgery. There were 952 studies relevant to POP surgery, 945 studies relevant to SUI surgery, 109 studies assessing surgery for POP and SUI combined and 73 studies relevant to mesh complication surgery. Combined POP/SUI studies included patients being treated for both conditions, as well as SUI and POP patients who were assessed together. While all study categories may have included papers that evaluated pelvic mesh surgery‐related outcomes, only mesh complication studies assessed outcomes of surgery to correct or remove previously implanted mesh.

Each study used a single measure or a combination of several measures. All extracted measures assessed subjective outcomes, but not all of these were PROMs/PREMs with evidence of psychometric testing. Roughly three quarters of studies (*n* = 1607, 77%) used a validated instrument; the remaining studies used an unvalidated or unspecified measure only (*n* = 472, 23%). Approximately a third of studies (*n* = 668, 32%) used both a validated instrument and an unvalidated or unspecified measure (Figure S1).

Five hundred and twenty‐two studies (25%) used a single PROM to assess outcomes, while 1082 (52%) used two or more measures. The maximum number of measures used in any study was ten (Figure S2).

### 3.2 Categorisation of Instruments

During data extraction, 151 PROMs/PREMs with evidence of psychometric testing were identified. The PROMs were categorised by scope, type of measurement and by outcome relevant to this review. There were 19 generic PROMs, which may be universally applied regardless of patient condition (Table S2), and 79 condition‐specific PROMs, designed to assess a particular condition or disease, such as urinary incontinence or obstructive defaecation (Table S3). Fifteen PROMs apply a preference‐based scale, used to calculate quality‐adjusted life years (QALYs) in health economic evaluations (Table S4). PROMs categorised by outcome included surgery‐specific PROMs (*n* = 17) (Table S5), sex‐specific PROMs (*n* = 8) (Table S6), mental health‐specific PROMs (*n* = 8) (Table S7) and body image‐specific PROMs (*n* = 4) (Table S8). Finally, three PREMs were extracted, which assess the impact of the process of care on patient experiences (Table S9).

Full details of all PROMs in each category are provided in Tables [Link], [Link], [Link], [Link], [Link], [Link], [Link], [Link].

### 3.3 Application of Instruments

**FIGURE 2:**
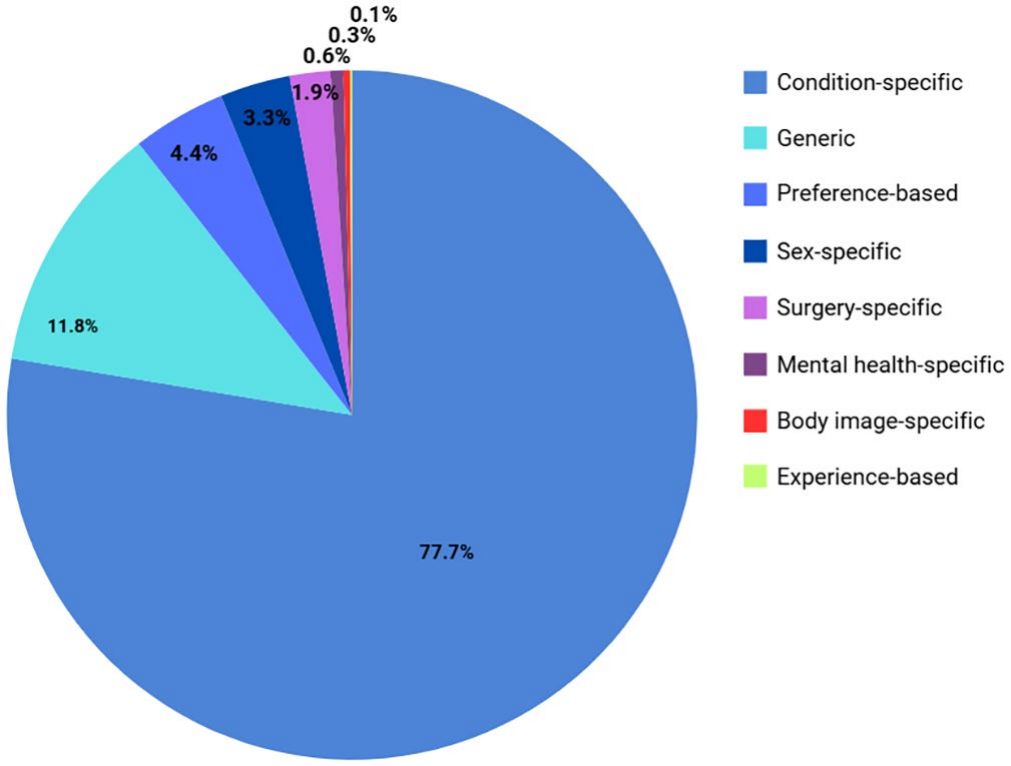
Number of applications of each PROM category across all studies.

Figure 2 shows the number of applications of each category of PROM across all study types. Condition‐specific PROMs were the most frequently used measures, applied 2800 times across all studies. Generic PROMs were used a total of 426 times, followed by preference‐based PROMs, applied 157 times and sex‐specific PROMs, applied 118 times. PROMs assessing surgery, mental health and body image were used 67, 20 and 11 times, respectively. PREMs were only applied three times across all studies.

**FIGURE 3:**
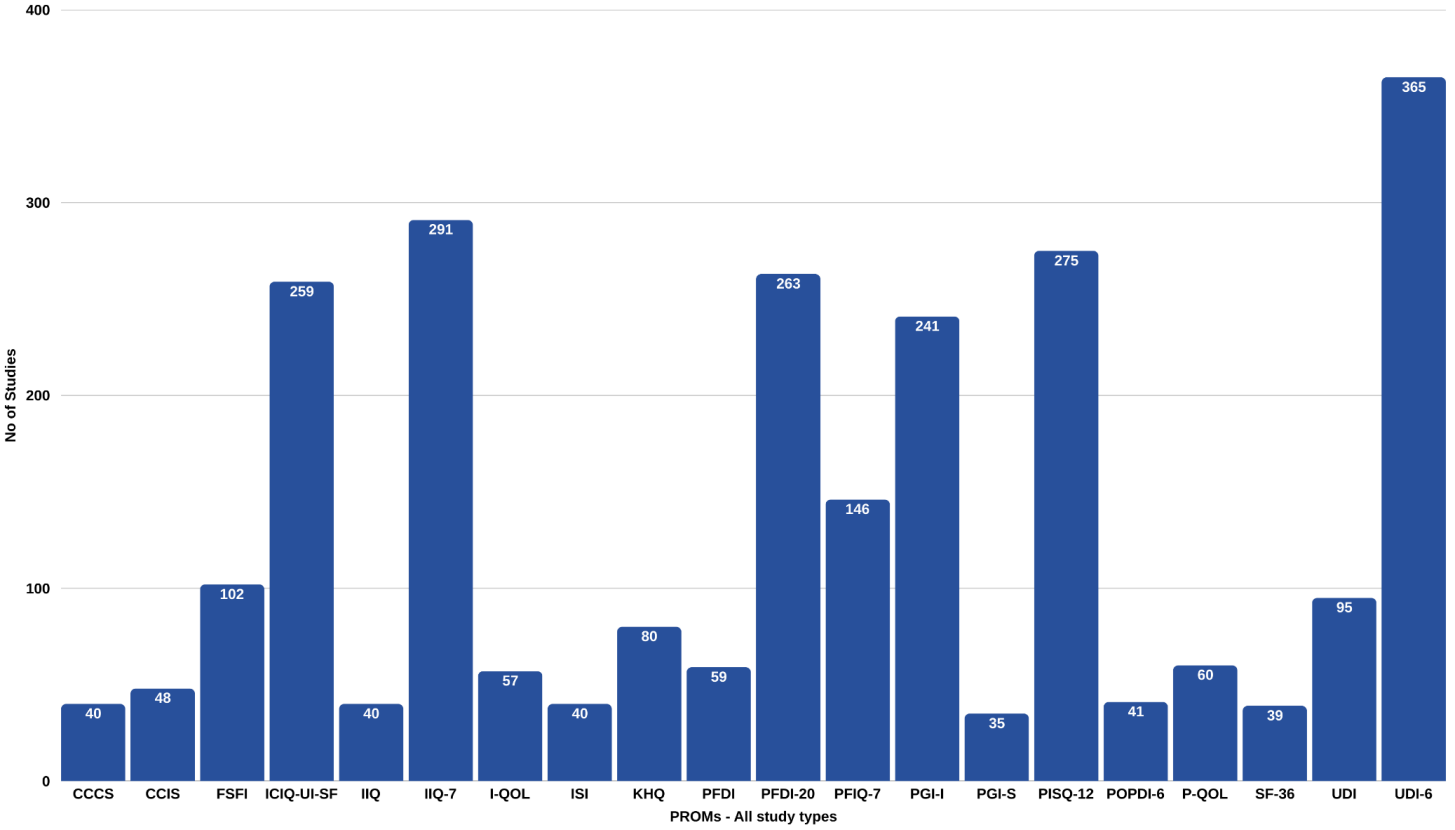
20 most frequently used PROMs across all study types.

Figure 3 provides the most commonly used PROMs. The majority of these are condition‐specific, evaluating subjective reports of the following symptoms and, to varying degrees, symptom effects on HRQL: urinary (UDI; UDI‐6; KHQ; ISI; I‐QOL; IIQ; IIQ‐7; ICIQ‐UI‐SF), bowel (CCCS; CCIS), prolapse (P‐QOL; POPDI‐6) and pelvic floor dysfunction (PFDI‐20; PFIQ‐7). The most prevalent generic measures were the single‐item PGI‐I and PGI‐S, assessing perceived improvement and condition severity, respectively. The PISQ‐12, which measures sexual function in patients with female pelvic floor disorders, and the generic FSFI were also frequently applied.

### 3.4 Pelvic Organ Prolapse

One hundred and twenty validated instruments were used to assess outcomes in POP studies. Figure S3 illustrates which of these were most used. The condition‐specific PFDI‐20, measuring the degree of bother associated with pelvic floor symptoms, was applied in over a quarter of all POP primary studies (*n* = 246). It comprises three subscales relating to prolapse (POPDI‐6), colorectal‐anal distress (CRADI‐8), and urinary symptoms (UDI‐6), which were also independently highly cited in POP studies (as well as their earlier versions e.g., UDI). The PFIQ‐7, an associated condition‐specific PROM measuring the effect of prolapse, bowel and urinary symptoms on physical function, social activities and mental health, was applied in 136 studies. Also used in a significant number of studies were the condition‐specific PISQ‐12 (*n* = 190) and the generic PGI‐I (*n* = 150). Other PROMs that were highly cited in POP studies assess constipation (CCCS; ODS), faecal incontinence (CCIS), urinary incontinence (IIQ‐7; ICIQ‐UI‐SF) and global HRQL (SF‐36; EQ‐5D measures). Ninety‐four PROMs were applied in fewer than ten POP studies each.

### 3.5 Stress Urinary Incontinence

In SUI studies, 75 instruments were used to assess surgical outcomes. The 20 most prevalent of these are shown in Figure S4. The majority of these were condition‐specific measures assessing urinary symptoms. As with POP studies, the generic PGI‐I was frequently applied (*n* = 157). The remaining PROMs used in SUI studies assess sexual function (PISQ‐12, *n* = 70; FSFI, *n* = 40), pelvic floor HRQL (PFIQ‐7, *n* = 15), global impression of condition severity (PGI‐S, *n* = 32) and global HRQL (EQ‐5D measures, *n* = 24). Fifty‐eight PROMs were each applied in less than ten SUI studies.

### 3.6 Combined POP/SUI

Fifty‐one PROMs were used in combined POP/SUI studies. As with the study types above, the condition‐specific UDI‐6, IIQ‐7, ICIQ‐UI‐SF and PFDI‐20 were popular measures, as well as the generic PGI‐I and sex‐specific FSFI and PISQ‐12.

### 3.7 Mesh Complications

Forty‐one PROMs were used in studies assessing mesh complication surgery. The PROMs most frequently applied were the generic PGI‐I (*n* = 20), condition‐specific UDI‐6 (*n* = 19), PFDI‐20 (*n* = 8), IIQ‐7 (*n* = 7) and the sex‐specific FSFI (*n* = 6). The remaining PROMs were each used in 5 or fewer primary studies.

### 3.8 Outcomes and Experiences

The outcomes assessed by each instrument were categorised and recorded—see Table S10 for details.

**FIGURE 4:**
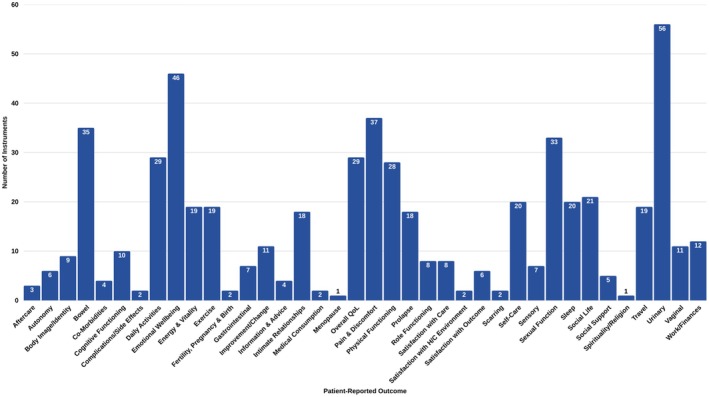
Numbers of instruments assessing each outcome.

Figure 4 shows the range of outcomes identified in this review and the numbers of PROMs that assess them. Roughly a third of extracted PROMs assess urinary symptoms (*n* = 56) and emotional wellbeing (*n* = 46), while around a fifth measure pain and discomfort (*n* = 37), bowel symptoms (*n* = 35), sexual function (*n* = 33) and physical functioning (*n* = 28), respectively. Some quality‐of‐life impacts are widely measured, for example, social life (*n* = 21), self‐care (*n* = 20), exercise (*n* = 19) and travel (*n* = 19). Other outcomes scarcely measured by these instruments collectively include role functioning (*n* = 8), social support (*n* = 5) and impact to spirituality/religion (*n* = 1). Additionally, few PROMs explicitly assess body image/identity (*n* = 9) or surgery‐specific outcomes, such as perception of scars (*n* = 2) or complications and side effects (*n* = 2). Patient‐reported experiences are also measured in a minority of measures: satisfaction with outcome is assessed by six instruments, information and advice by four and aftercare by three.

Figure S5 depicts the percentages of instruments by the number of outcomes they measure. Half of all PROMs (*n* = 74) identified in this review assess a single outcome. A third of these PROMs are specific to urinary symptoms only (*n* = 23). Other issues that are assessed by single‐outcome PROMs include emotional wellbeing (*n* = 9), bowel symptoms (*n* = 8), pain and discomfort (*n* = 8), sexual function (*n* = 4), prolapse (*n* = 3) and global HRQL (*n* = 3).

Thirty‐three PROMS measure between two and five outcomes and 31 measure between six and nine outcomes. Thirteen PROMs measure ten or more outcomes. These include the generic PROMs SIP, WHOQoL‐BREF and QS‐F (sex‐specific), PSR‐15, QoR‐15 and QoR‐40 (all surgery‐specific), as well as the preference‐based PROMs 15D, SF‐12 and SF‐36. The four most broad‐spectrum condition‐specific PROMs are the ePAQ‐PF (pelvic floor dysfunction), the ICIQ‐LUTSqol (lower urinary tract symptoms), the KHQ (urinary incontinence) and the P‐QOL (prolapse).

### 3.9 Items, Recall Periods and Response Options

The mean number of core items across all PROMs/PREMs is 18 (SD = 21); the median is 12 (IQR = 7, 21). The number of items ranged from 1 (e.g., PGI‐C, PGI‐I, PGI‐S) to 156 (e.g., ePAQ‐PF). Figure S6 provides the mean number of core items for each instrument category.

Fifty‐five per cent of the measures used Likert scales only to record patient responses (*n* = 83). Likert scales were predominantly used to assess severity (e.g., PGI‐S, POPDI‐6, PAC‐SYM), improvement (e.g., PGI‐I, PGI‐C) and frequency (e.g., ODS, KHQ). Other scales used included numerical rating scales (e.g., QoR‐15, QoR‐14), visual analogue scales (e.g., EQ‐VAS), dichotomous (Yes/No) response options (e.g., ADL), count data (e.g., IPAQ), nominal/categorical options (e.g., MPQ, SIP) and image scales (e.g., Wong Baker Pain Scale). Thirty per cent of PROMs used a combination of scales, and eight included free text response options (e.g., ePAQ‐PF).

PROM recall periods included current perception (16%, *n* = 24), 24 h (3%, *n* = 5), 1 week (6%, *n* = 9), 2 weeks (4%, *n* = 6), 4 weeks/1 month (21%, *n* = 32), 3 months (12%, *n* = 18) and 6 months (1%, *n* = 2). Forty‐seven measures did not specify a recall period (31%). Six instruments referred to ‘most recent surgery’ or the ‘since the start of treatment’.

All details relating to items, recall periods and response options can be found in Tables [Link], [Link], [Link], [Link], [Link], [Link], [Link], [Link].

## 4 Discussion

### 4.1 Main Findings

This review identified 2079 primary studies that used a subjective measure to assess patient‐reported outcomes and experiences relating to surgery for POP, SUI or mesh complications. The majority of studies collected PRO data for prolapse or stress incontinence surgery, while relatively few assessed outcomes relating to mesh complication surgery. Seventy‐seven per cent of the included studies used a validated PROM.

One hundred and fifty‐one validated PROMs/PREMs were identified and included in data extraction. The PROMs that were most used tended to be condition‐specific, assessing pelvic floor disorders and, in some cases, chronic or acute illnesses and co‐morbidities. Generic PROMs such as the Patient Global Impression measures were frequently used, as well as preference‐based tools, such as the RAND measures (SF‐12; SF‐36) and EQ‐5D scales. The PISQ‐12 and FSFI were popular measures for assessing sexual function. PROMs specific to surgery, mental health and body image were used to a lesser extent, and there was negligible use of standalone PREMs, such as the CSQ, which measures consumer satisfaction with health services.

In addition to the heterogeneity of the instruments utilised across the surgical studies, there is significant variation in the outcomes that are measured by these tools. In order to compare the PROMs, a categorisation of outcomes was undertaken as part of this review (Table S10), demonstrating an overall emphasis among the instruments on symptom impacts, as well as emotional wellbeing, pain and sexual function. Some HRQL impacts (e.g., social life and travel) are also frequently measured. Other HRQL outcomes (e.g., social support), surgery‐specific outcomes (e.g., scarring, complications and side‐effects), and patient healthcare experiences are not evaluated by many measures. The majority of PROMs were narrow in scope, with roughly two thirds assessing one to three outcomes only (Figure S5). A few broad‐spectrum/comprehensive PROMs were identified, but these were not highly cited. None of the identified PROMs measured all PROs relevant to patients undergoing surgery for POP, SUI or mesh complications.

While most of the measures used were validated PROMs, a significant number of the included studies also or only used unvalidated researcher‐developed questions or tools (Figure S1), which further suggests that existing PROMs may not be sufficient for capturing all relevant PRO data. While an appraisal of the psychometric properties of the extracted PROMs was beyond the scope of this review, elsewhere concerns have been reported around the utilisation of PROMs with insufficient psychometric evidence [4, 35], a lack of validation data for the use of PROMs in specific populations or settings [36], and the use of measures not originally intended for clinical practice [37]. Most of the PROMs identified in this review were not developed to assess the specific surgical outcomes and experiences of all three patient groups (POP, SUI and mesh complications). In addition, the few surgery‐specific PROMs that were identified were not widely used and either assessed relatively few outcomes or were not specific to pelvic floor disorders.

A challenge for clinicians and researchers is choosing an appropriate PROM from the many that are available, particularly in light of the lack of consensus regarding which instrument(s) should be used [5]. The instrument choices of the studies included in this review may have been informed by the current approach proposed by The International Consultation on Incontinence Questionnaire (ICIQ) [7, 38] and The Pelvic Floor Disorders Consortium (PFDC) [10] to use a battery of validated instruments to collect PRO data, as well as conventions around using generic and condition‐specific measures concurrently [37, 39]. The majority of studies used a combination of PROMs, with some using as many as ten different instruments (Figure S2). Furthermore, the measures most highly rated by the ICIQ and PFDC for their psychometric properties, brevity and ease of use [7, 10, 38] were among the most cited in this review (e.g., the ICIQ tools, UDI‐6, PFDI‐20).

PROMs are often selected for brevity [10] due to the association of longer PROMs with participant response burden, the risk of high rates of missing data [40, 41, 42], and practical concerns around clinical use [10, 36]. The findings of this review reflect this: the mean number of core items for the 20 most cited PROMs is 15 (SD = 14, range 1–46), lower than the average for the full range of instruments. Meanwhile, the longest of the extracted PROMs, the ePAQ‐PF and SIP, were applied in few studies by comparison. However, given that pelvic floor disorders such as urinary incontinence, pelvic organ prolapse, sexual dysfunction, obstructive defecation and accidental bowel leakage often overlap and occur concurrently, PROMs in this area need to be broad enough in scope to make a comprehensive assessment of patients' experiences of all their conditions. Some researchers have argued that the length of a PROM may not necessarily increase response burden [43, 44] and that this should not outweigh the need to assess outcomes that are important to patients [41]. Importantly, the use of several measures to capture all PRO concepts of interest in one setting [42, 45] may also increase completion times and pose difficulties for researchers in assimilating the results of different instruments [46], as well as being longer than existing comprehensive PROMs to complete.

### 4.2 Strengths and Limitations

To our knowledge, this is the first scoping review to provide a comprehensive inventory of the PROMs/PREMs that have been used in this surgical population and reported in published peer‐reviewed literature. Not only will this inform the development of the NIHR‐funded APPRAISE PROM [19], but this review provides an important resource for clinicians and researchers to enable PROM comparison and selection, with all the data collected being available in repositories. The inability to assess the methodological quality of included studies and the psychometric properties of the extracted instruments is a limitation of this review. This level of evaluation was prevented by the number of studies identified. However, further systematic reviews are being undertaken by the APPRAISE team, which will include rigorous psychometric assessment of PROMs used in specific surgical patient groups (e.g., mesh complications), following COSMIN guidelines. An additional limitation relates to the inclusion of papers reported in English only. Non‐English language PROMs have been included in Table S11, but without validated translations, it was not possible to include them in the synthesis. Finally, this review does not provide an exhaustive list of all urogynaecological PROMs, only those used in patients undergoing SUI, POP or mesh complication surgery. Studies that use PROMs to assess outcomes not specific to surgery were excluded. Newer PROMs or PROMs in development will also not have been identified if they have not yet been applied in published primary studies.

### 4.3 Interpretation

Our review identified a range of PROMs developed specifically for pelvic floor disorders as well as generic and preference‐based PROMs applicable across populations. The former cover a range of impacts to patients' health and quality of life, with many focusing on symptom specific outcomes. Given that outcomes relevant to women undergoing surgery for pelvic floor disorders span multiple domains of HRQL, it is possible that symptom‐specific outcome measures may not provide a comprehensive overview of the effects of surgery. The application of multiple measures may improve comprehensiveness, but heterogeneity of instrument use may pose challenges in comparing outcomes in this population of women. In addition, inconsistencies in outcome data collection and reporting for POP and SUI surgical interventions trials can produce results that are difficult to compare, preventing effective data synthesis and quality meta‐analyses [47]. In order to promote higher quality evidence, the development of ‘core outcome sets’ (COS) and ‘core outcome measures sets’ (COMS) is essential. These efforts have been standardised by the Core Outcome Measures in Effectiveness Trials (COMET) initiative and COS have been developed in many areas of research as well as clinical practice [48]. In the field of pelvic floor research and practice, CHORUS [49] is an international group working towards the development of such COS and COMS [12, 13, 14, 15, 16].

Previous recommendations [4] highlight the need for a comprehensive tool to be considered in place of a battery of measures for the reasons outlined above. Currently, there is no standardised instrument available. To assess all of the relevant outcomes identified in this review, a new single surgery‐specific PROM will need to be comprehensive. This may yield a higher number of core items, but the burden of completion on patients can be mitigated by the need to only complete one measure that is highly relevant to their experiences. This would also enable greater ease in comparing outcomes through the use of a consistent scoring system, while linking the new PROM to a registry of completed surgical procedures (a recommendation of the Cumberledge Review, *First Do No Harm* [17]) would allow necessary evidence to be collected to inform best treatment decisions. As part of the development of the new APPRAISE PROM, further work is currently underway to identify all of the relevant outcomes for this population, especially for those undergoing mesh complication surgery for which there are fewer existing studies and population‐specific PROMs available. This research is also assessing whether PROMs are sensitive to cultural differences in HRQL impacts and to diverse populations (including LGBTQIA+ patients).

## 5 Conclusion

The effective treatment of pelvic floor disorders relies on assessment of patient‐reported symptoms and validated instruments to provide reliable and robust PRO data in both clinical and research settings. This review included 2079 primary studies using subjective measures to assess outcomes relevant to POP, SUI or mesh corrective surgery. An evaluation of outcomes measured by the 151 extracted PROMs and PREMs indicates that currently no existing instrument collects all PRO data relevant to these three surgical patient groups. The findings of this review will inform the conceptual framework of a new surgery‐specific PROM developed by the NIHR‐funded APPRAISE team. Further research is required on the standardisation of outcomes to better understand patient experiences.

## Author Contributions

G.L.J. and A.M.B. conceived the idea. All authors were involved in the design of the review. G.F. and K.B. designed the search strategy and carried out the literature searches. G.F., K.B., A.M.B. and V.F. collected the data. G.F. led the analysis. All authors were involved with the analysis and interpretation of the data. G.F. drafted the article with input from all other authors. All authors critically reviewed the article and approved the final version for publication.

## Conflicts of Interest

The authors declare no conflicts of interest.

## Supporting information


**Appendix S1:**PRISMA Checklist.

**Appendix S2:** Detailed Search Strategy.

**Appendix S3:** List of acronyms.

**Figure S1:** Clustered bar chart—Count of study type by type of subjective measure used.

**Figure S2:** Bar chart—Number of measures applied in each study.

**Figure S3:** Bar chart—Twenty most used instruments in primary studies assessing POP surgery.

**Figure S4:** Bar chart—Twenty most used instruments in primary studies assessing SUI surgery.

**Figure S5:** Pie chart—Percentages of PROMs by the number of outcomes measured.

**Figure S6:** Bar chart—Mean number of items in each category of instrument.

**Table S1:** Included Studies.

**Table S2:** Table of generic PROMs—extracted data.

**Table S3:** Table of condition‐specific PROMs—extracted data.

**Table S4:** Table of preference‐based PROMs—extracted data.

**Table S5:** Table of surgery‐specific PROMs—extracted data.

**Table S6:** Table of sex‐specific PROMs—extracted data.

**Table S7:** Table of mental health‐specific PROMs—extracted data.

**Table S8:** Table of body image‐specific PROMs—extracted data.

**Table S9:** Table of PREMs—extracted data.

**Table S10:** Table of outcomes assessed by each instrument.

**Table S11:** Table of non‐English language PROMs.

## Data Availability

The data that support the findings of this study are available from the corresponding author upon reasonable request.
