# Supplementary material for: Patient‐Reported Outcome Measures Used to Assess Surgical Interventions for Pelvic Organ Prolapse, Stress Urinary Incontinence and Mesh Complications: A Scoping Review for the Development of the APPRAISE PROM
Source: BJOG. 2025 Sep 24;133(2):218–27. doi: 10.1111/1471-0528.18355 (PMC12678042; doi:10.1111/1471-0528.18355)
Supplement: Supplementary file 2 — Appendix S2: Detailed Search Strategy. [file BJO-133-218-s014.docx]

**Appendix S2: Detailed Search Strategy**

Example given is for MEDLINE database (EBSCO host)

1. Condition

| S1 | Keywords | TI (incontinence OR “stress urinary incontinence” OR “stress incontinence” OR “SUI” OR “pelvic floor disorder*” OR “pelvic floor dysfunction” OR cystocele OR rectocele OR enterocele OR “pelvic organ prolapse*” OR “POP” OR “vaginal prolapse” OR "vaginal wall prolapse*" OR “uterine prolapse” OR “uterovaginal prolapse” OR “rectal prolapse” OR “mesh complication*”) OR AB (incontinence OR “stress urinary incontinence” OR “stress incontinence” OR “SUI” OR “pelvic floor disorder*” OR “pelvic floor dysfunction” OR cystocele OR rectocele OR enterocele OR “pelvic organ prolapse” OR “POP” OR “vaginal prolapse” OR "vaginal wall prolapse" OR “uterine prolapse*” OR “uterovaginal prolapse” OR “rectal prolapse” OR “mesh complication*” ) |
| --- | --- | --- |
| S2 | Mesh Headings | (MH "Surgical Mesh") OR (MH "Urinary Incontinence+") OR (MH "Urinary Incontinence, Stress") OR (MH "Pelvic Floor Disorders") OR (MH "Cystocele") OR (MH "Rectocele") OR (MH "Pelvic Organ Prolapse") OR (MH "Uterine Prolapse") OR (MH "Rectal Prolapse") |
| S3 |  | S1 OR S2 |

1. Intervention

| S4 | Keywords | TI ( “surgical trial*” OR “vaginal N5 surger*” OR “vaginal N5 surgical” OR “surgery for vaginal” OR “vaginal surger*” OR "prolapse N5 surger*” OR “prolapse N5 surgical” OR “surgery for prolapse” OR “prolapse surger*” OR “stress urinary incontinence N5 surger*” OR “stress urinary incontinence N5 surgical” OR “surgery for stress urinary incontinence” OR “stress urinary incontinence surger*” OR “urinary incontinence N5 surger*” OR “urinary incontinence N5 surgical” OR “surgery for urinary incontinence” OR “urinary incontinence surger*” OR “incontinence N5 surger*” OR “incontinence N5 surgical” OR “surgery for incontinence” OR “incontinence surger*” OR "SUI N5 surger*" OR "surgical N5 SUI" OR “surgery for SUI” OR “SUI surger*” OR "POP N5 surger* OR "surgical N5 POP" OR “surgery for POP” OR “POP surger*” OR "UI N5 surger*" OR "surgical N5 UI" OR “surgery for UI” OR “UI surger*” OR “vaginal mesh” OR "transvaginal repair" OR “transvaginal mesh” OR “tension-free vaginal tape” OR transobturator OR “mesh removal” OR “mesh repair” OR “pelvic mesh” OR “mesh N5 surger*” OR “surgery for mesh” OR “mesh implant*” OR hysterectom* OR “vaginal hysterectom*” OR “abdominal hysterectom*” OR “pelvic floor procedure*” OR “pelvic N5 surger*” OR “surgery for pelvic” OR “vaginal wall repair” OR “vaginal repair” OR “paravaginal repair” OR “anterior colporrhaphy” OR “sacrospinous fixation” OR “Manchester repair” OR “Manchester procedure” OR “sacrospinous hysteropexy” OR “perineal surgery” OR “levator ani plication” OR colpocleisis OR sacrocolpopexy OR sacrocervicopexy OR “uterosacral ligament suspension” OR “sacrospinous ligament fixation” OR sacrohysteropexy OR hysteropexy OR “anterior abdominal wall hysteropexy” OR “uterosacral hysteropexy” OR “sacrospinous hysteropexy” OR colposuspension OR “fascial sling” OR “pubovaginal sling” OR “sub-urethral sling” OR “suburethral sling” OR “mid-urethral sling” OR “midurethral sling” OR “artificial urinary sphincter” OR “urethral bulking” OR rectopexy) OR AB ( “surgical trial*” OR “vaginal N5 surger*” OR “vaginal N5 surgical” OR “surgery for vaginal” OR “vaginal surger*” OR "prolapse N5 surger*” OR “prolapse N5 surgical” OR “surgery for prolapse” OR “prolapse surger*” OR “stress urinary incontinence N5 surger*” OR “stress urinary incontinence N5 surgical” OR “surgery for stress urinary incontinence” OR “stress urinary incontinence surger*” OR “urinary incontinence N5 surger*” OR “urinary incontinence N5 surgical” OR “surgery for urinary incontinence” OR “urinary incontinence surger*” OR “incontinence N5 surger*” OR “incontinence N5 surgical” OR “surgery for incontinence” OR “incontinence surger*” OR "SUI N5 surger*" OR "surgical N5 SUI" OR “surgery for SUI” OR “SUI surger*” OR "POP N5 surger* OR "surgical N5 POP" OR “surgery for POP” OR “POP surger*” OR "UI N5 surger*" OR "surgical N5 UI" OR “surgery for UI” OR “UI surger*” OR “vaginal mesh” OR "transvaginal repair" OR “transvaginal mesh” OR “tension-free vaginal tape” OR transobturator OR “mesh removal” OR “mesh repair” OR “pelvic mesh” OR “mesh N5 surger*” OR “surgery for mesh” OR “mesh implant*” OR hysterectom* OR “vaginal hysterectom*” OR “abdominal hysterectom*” OR “pelvic floor procedure*” OR “pelvic N5 surger*” OR “surgery for pelvic” OR “vaginal wall repair” OR “vaginal repair” OR “paravaginal repair” OR “anterior colporrhaphy” OR “sacrospinous fixation” OR “Manchester repair” OR “Manchester procedure” OR “sacrospinous hysteropexy” OR “perineal surgery” OR “levator ani plication” OR colpocleisis OR sacrocolpopexy OR sacrocervicopexy OR “uterosacral ligament suspension” OR “sacrospinous ligament fixation” OR sacrohysteropexy OR hysteropexy OR “anterior abdominal wall hysteropexy” OR “uterosacral hysteropexy” OR “sacrospinous hysteropexy” OR colposuspension OR “fascial sling” OR “pubovaginal sling” OR “sub-urethral sling” OR “suburethral sling” OR “mid-urethral sling” OR “midurethral sling” OR “artificial urinary sphincter” OR “urethral bulking” OR rectopexy) |
| --- | --- | --- |
| S5 | Mesh Headings | (MH "Hysterectomy") OR (MH "Hysterectomy, Vaginal") OR (MH "Suburethral Slings") |
| S6 |  | S4 OR S5 |

1. Outcomes – PROMs/PREMs

| S7 | Keywords | TI (PROM* OR PRO OR “Patient reported outcome*” OR "patient reported outcome measure*" OR PREM* OR “patient reported experience*” OR “self-complete*” OR “self-administer*” OR “self-report*” OR “self-assess*” OR “patient reported” OR “subjective*” OR “quality of life” OR “health related quality of life” OR qol OR hrqol OR “hr qol” OR severity OR satisfaction OR “outcome measure*” OR questionnaire* OR survey* OR inventory OR inventories OR index* OR scale* OR instrument* OR score* OR tool* OR “rating scale*” OR “health status*” OR assessment*) OR AB (PROM* OR PRO OR “Patient reported outcome*” OR "patient reported outcome measure*" OR PREM* OR “patient reported experience*” OR “self-complete*” OR “self-administer*” OR “self-report*” OR “self-assess*” OR “patient reported” OR “subjective*” OR “quality of life” OR “health related quality of life” OR qol OR hrqol OR “hr qol” OR severity OR satisfaction OR “outcome measure*” OR questionnaire* OR survey* OR inventory OR inventories OR index* OR scale* OR instrument* OR score* OR tool* OR “rating scale*” OR “health status*” OR assessment*) |
| --- | --- | --- |
| S8 | Mesh Headings | (MH "Quality of Life") OR (MH "Self Report") OR (MH "Patient Reported Outcome Measures") OR (MH "Patient Health Questionnaire") OR (MH "Surveys and Questionnaires") |
| S9 |  | S7 OR S8 |

1. Study design

| S10 | Keyword | TI (perioperative OR post-operative* OR postoperative* OR “follow up” OR prospective OR cohort OR validat* OR review OR RCT* OR “random* controlled trial*” OR “randomised trial*” OR "randomized trial" OR longitudinal OR “cross-sectional” OR retrospective) OR AB (perioperative OR post-operative* OR postoperative* OR “follow up” OR prospective OR cohort OR validat* OR review OR RCT* OR “random* controlled trial*” OR “randomised trial*” OR "randomized trial" OR longitudinal OR “cross-sectional” OR retrospective) |
| --- | --- | --- |

| S11 | Search command | S3 AND S6 AND S9 AND S10 |
| --- | --- | --- |

1. Male terms to exclude

| S12 | Keyword | (TI (erectile OR "adjustable transobturator male system" OR "adjustable trans obturator male system" OR “post prostatectomy" OR "post-prostatectomy" OR postprostatectomy OR “male sling” OR "transobturator-retrourethral" OR prostatectomy) OR AB (erectile OR "adjustable transobturator male system" OR "adjustable trans obturator male system" OR “post prostatectomy" OR "post-prostatectomy" OR postprostatectomy OR “male sling” OR "transobturator-retrourethral" OR prostatectomy)) |
| --- | --- | --- |

| S13 | Search command | S11 NOT S12 |
| --- | --- | --- |
