# Supplementary material for: Patient‐Reported Outcome Measures Used to Assess Surgical Interventions for Pelvic Organ Prolapse, Stress Urinary Incontinence and Mesh Complications: A Scoping Review for the Development of the APPRAISE PROM
Source: BJOG. 2025 Sep 24;133(2):218–27. doi: 10.1111/1471-0528.18355 (PMC12678042; doi:10.1111/1471-0528.18355)
Supplement: Supplementary file 3 — Appendix S3: List of acronyms. [file BJO-133-218-s011.docx]

List of Acronyms

| 15D | 15-Dimensional Health-Related Quality of Life Measure |
| --- | --- |
| ADL | Activities of Daily Living (aka Katz’s Index of Independence in Activities of Daily Living; KATZ ADL) |
| APFQ | Australian Pelvic Floor Questionnaire (aka Queensland Female Pelvic Floor Questionnaire) |
| APPRAISE | A Patient-reported outcome measure for PRolApse, Incontinence and meSh complication surgery |
| CCCS | Cleveland Clinic Constipation Scoring System (aka Wexner Score for Constipation / Wexner-Agachan Constipation Scoring System/Wexner Score for Obstructive Defaecation) |
| CCIS | Cleveland Clinic Incontinence Scoring System (aka Wexner Score for Faecal Incontinence / Cleaveland Clinic Florida Faecal Incontinence Score (CCF-FIS)) |
| COMET | Core Outcome Measures in Effectiveness Trials |
| COMS | Core outcome measures sets |
| COS | Core outcome sets |
| CRADI-8 | Colorectal Anal Distress Inventory, Short Form |
| CSQ | Client Satisfaction Questionnaire |
| ePAQ-PF | Electronic Personal Assessment Questionnaire - Pelvic Floor |
| EQ-5D | EuroQol 5 level |
| EQ-VAS | EuroQol Visual Analogue Scale |
| FSFI | Female Sexual Function Index |
| HRQL | Health-related quality of life |
| ICIQ | The International Consultation on Incontinence Questionnaire |
| ICIQ-LUTSqol | ICIQ Lower Urinary Tract Symptoms - Quality of Life Module |
| ICIQ-UI-SF | ICIQ Urinary Incontinence, Short Form |
| IIQ | Incontinence Impact Questionnaire |
| IIQ-7 | Incontinence Impact Questionnaire, Short Form |
| IPAQ | International Physical Activity Questionnaire |
| I-QOL | Incontinence Quality of Life Instrument |
| ISI | Incontinence Severity Index |
| KHQ | Kings Health Questionnaire |
| LGBTQIA+ | Lesbian, gay, bisexual, transgender, queer, questioning, intersex, asexual + |
| MPQ | McGill Pain Questionnaire |
| MUI | Mixed urinary incontinence |
| NICE | National Institute for Health and Care Excellence |
| NIHR | National Institute for Health and Care Research (UK) |
| ODS | Obstructed Defaecation Syndrome Score |
| PAC-SYM | Patient Assessment of Constipation Symptoms |
| PFD | Pelvic floor disorder |
| PFDI-20 | Pelvic Floor Distress Inventory (20 Item) |
| PFIQ-7 | Pelvic Floor Impact Questionnaire, Short Form |
| PGI-C | Patient Global Impression - Change |
| PGI-I | Patient Global Impression - Improvement |
| PGI-S | Patient Global Impression - Severity |
| PISQ-12 | Pelvic Organ Prolapse Urinary Incontinence Sexual Questionnaire, Short Form |
| POP | Pelvic organ prolapse |
| POPDI-6 | Pelvic Organ Prolapse Distress Inventory, Short Form |
| PPI | Patient and public involvement |
| P-QOL | Pelvic Organ Prolapse Quality of Life Instrument |
| PREM | Patient reported experience measure |
| PRISMA | Preferred Reporting Items for Systematic reviews and Meta-Analyses |
| PRO | Patient reported outcome |
| PROM | Patient reported outcome measure |
| PSR-15 | Postdischarge Surgical Recovery Scale - 15 Item |
| QoR-15 | Quality of Recovery, Short Form |
| QoR-40 | Quality of Recovery |
| QS-F | Quality of Sexual Function Questionnaire |
| SF-12 | 12-Item Short Form Survey (RAND) |
| SF-36 | 36-Item Short Form Survey (RAND) |
| SIP | Sickness Impact Profile |
| SUI | Stress urinary incontinence |
| UDI | Urinary Distress Inventory |
| UDI-6 | Urinary Distress Inventory, Short Form |
| WHOQoL-BREF | World Health Organization Quality of Life Assessment Instrument |
