# Supplementary figures and images for: Patient‐Reported Outcome Measures Used to Assess Surgical Interventions for Pelvic Organ Prolapse, Stress Urinary Incontinence and Mesh Complications: A Scoping Review for the Development of the APPRAISE PROM
Source: BJOG. 2025 Sep 24;133(2):218–27. doi: 10.1111/1471-0528.18355 (PMC12678042; doi:10.1111/1471-0528.18355)

**Figure S1: Count of study type by type of subjective measure used**


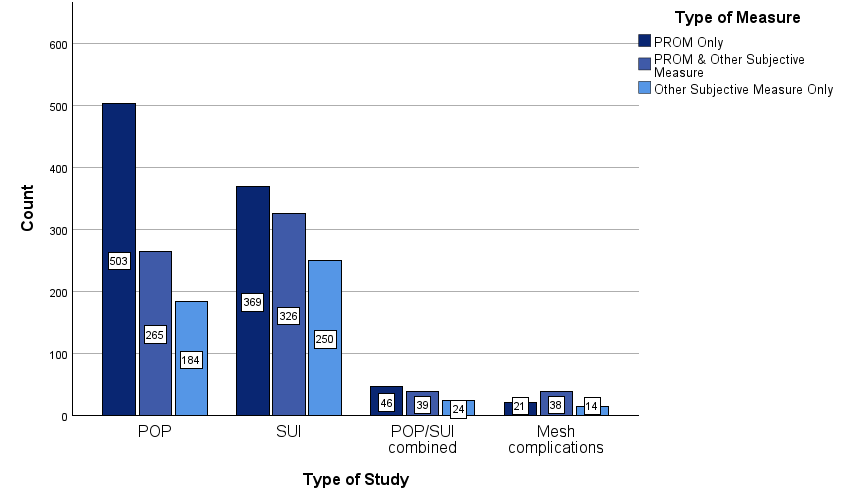

Supplement: Supplementary file 4 — Figure S1: Clustered bar chart—Count of study type by type of subjective measure used. [file BJO-133-218-s012.docx]

**Figure S2: Number of measures applied in each study**


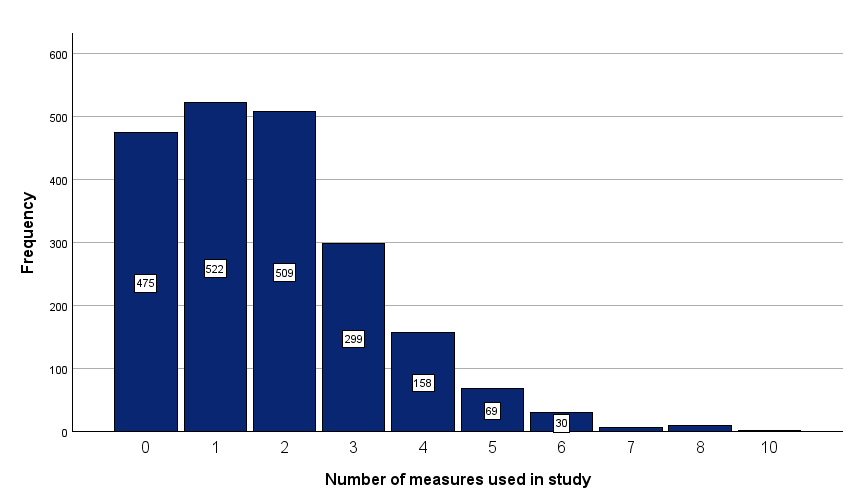

Supplement: Supplementary file 5 — Figure S2: Bar chart—Number of measures applied in each study. [file BJO-133-218-s009.docx]

**Figure S3: Twenty most used instruments in primary studies assessing POP surgery**


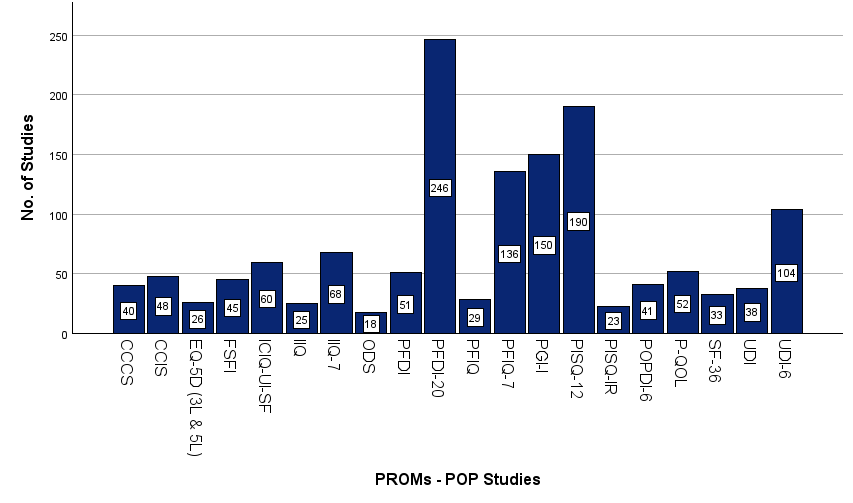

Supplement: Supplementary file 6 — Figure S3: Bar chart—Twenty most used instruments in primary studies assessing POP surgery. [file BJO-133-218-s003.docx]

**Figure S4: Twenty most used instruments in primary studies assessing SUI surgery**


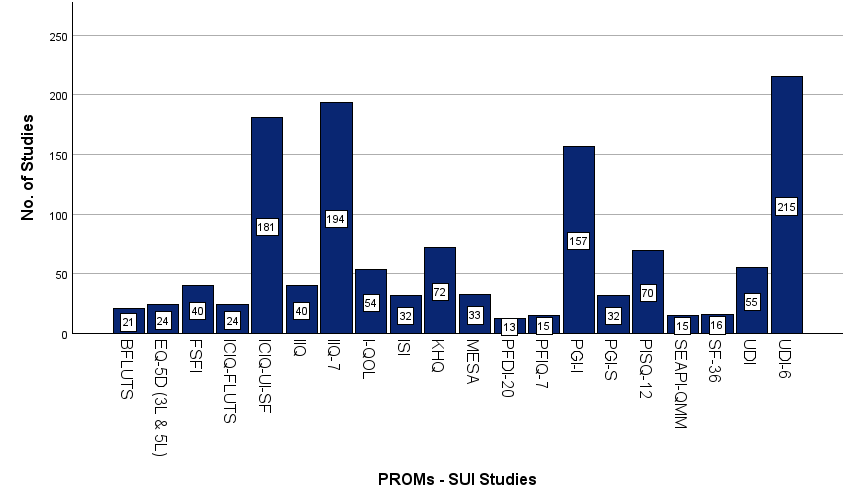

Supplement: Supplementary file 7 — Figure S4: Bar chart—Twenty most used instruments in primary studies assessing SUI surgery. [file BJO-133-218-s019.docx]

**Figure S5: Percentages of PROMs by the Number of Outcomes Measured**


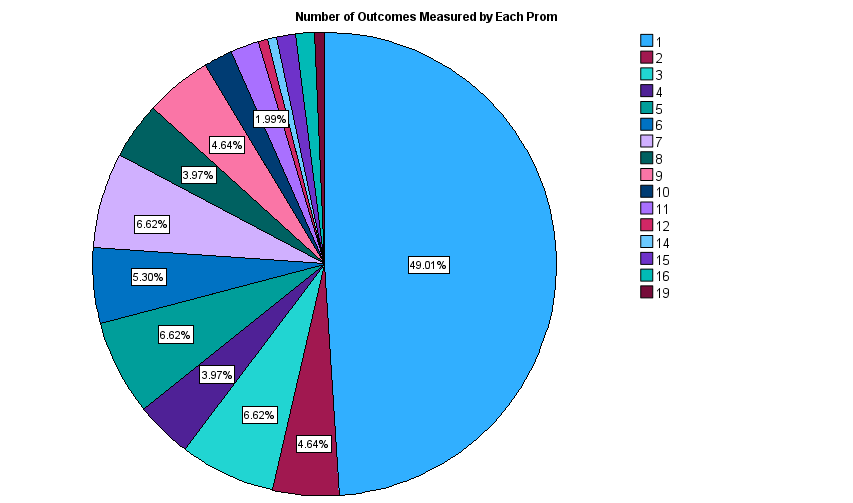

Supplement: Supplementary file 8 — Figure S5: Pie chart—Percentages of PROMs by the number of outcomes measured. [file BJO-133-218-s006.docx]

**Figure S6: Mean number of items in each category of instrument**


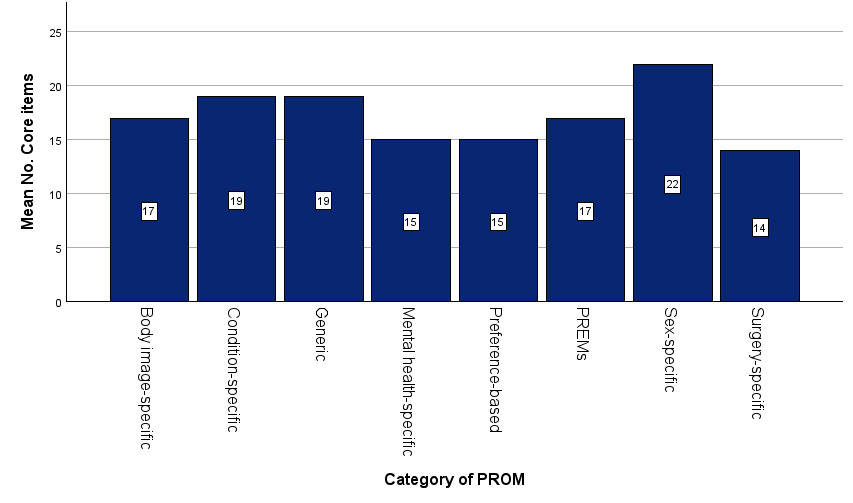

Supplement: Supplementary file 9 — Figure S6: Bar chart—Mean number of items in each category of instrument. [file BJO-133-218-s005.docx]
