# Supplementary material for: Patient‐Reported Outcome Measures Used to Assess Surgical Interventions for Pelvic Organ Prolapse, Stress Urinary Incontinence and Mesh Complications: A Scoping Review for the Development of the APPRAISE PROM
Source: BJOG. 2025 Sep 24;133(2):218–27. doi: 10.1111/1471-0528.18355 (PMC12678042; doi:10.1111/1471-0528.18355)
Supplement: Supplementary file 11 — Table S2: Table of generic PROMs—extracted data. [file BJO-133-218-s013.docx]

**Table S2: Generic PROMs- Extracted Data**

| **PROM (short title)** | **PROM**  **(long title)** | **Example study reporting psychometric properties** | **PROM Aim** | **No. Core items** | **No. Bother items** | **Type of Response Categories**** | **Recall Period** | **No. POP Studies** | **No. SUI Studies** | **No. POP/SUI Combined Studies** | **No. Mesh Studies** |
| --- | --- | --- | --- | --- | --- | --- | --- | --- | --- | --- | --- |
| **BIPQ** | Brief Illness Perception Questionnaire  Brief IPQ; B-IPQ* | ‌Broadbent et al. (2006). DOI: 10.1016/j.jpsychores.2005.10.020  ‌ | To rapidly assess the cognitive and emotional representations of illness | 9 | 0 | NRS, free text | Not specified | 1 | 0 | 0 | 0 |
| **DN4** | DN4 Pain Questionnaire | Bouhassira et al. (2005). DOI: 10.1016/j.pain.2004.12.010 | To detect neuropathic pain | 4 | 0 | Dichotomous | Current perception | 0 | 0 | 0 | 2 |
| **FAS** | Functional Activity Scale | Pfeffer et al. (1982). DOI: 10.1093/geronj/37.3.323 ‌ | To measure instrumental activities of daily living | 10 | 0 | Nominal | Not specified | 1 | 0 | 0 | 0 |
| **GHQ-12** | General Health Questionnaire | Hardy et al. (1999). DOI: 10.1037/1040-3590.11.2.159 | To detect psychiatric disorders in community settings and non-psychiatric clinical settings | 14 | 0 | Likert | Not specified | 0 | 0 | 1 | 0 |
| **Godin-Shephard** | The Godin-Shephard Leisure-Time Physical Activity Questionnaire | Godin (2011).  DOI: 10.14288/hfjc.v4i1.82 | To measure types of exercise undertaken by the respondent | 3 | 0 | Count data | 1 week | 1 | 0 | 0 | 0 |
| **IADL** | Instrumental Activities of Daily Living Scale | Lawton et al. (2020). DOI: 10.1093/geront/9.3_part_1.179 | To assess everyday functional competence | 8 | 0 | Nominal | Not specified | 1 | 0 | 0 | 0 |
| **IPAQ** | International Physical Activity Questionnaire | Craig et al. (2003). DOI: 10.1249/01.MSS.0000078924.61453.FB.‌ | To measure physical activity levels | 7 | 0 | Count data | 7 days | 0 | 3 | 0 | 0 |
| **LCB** | Locus of Control of Behaviour Scale | Craig et al. (1984). DOI: 10.1111/j.2044-8341.1984.tb01597.x | To measure the extent to which a person perceives events as being under their control | 17 | 0 | Likert | Not specified | 0 | 0 | 1 | 0 |
| **MPQ** | McGill Pain Questionnaire | Melzack (1975). DOI: 10.1016/0304-3959(75)90044-5 | To measure clinical pain | 28 | 0 | Nominal | Current perception | 0 | 1 | 0 | 1 |
| **MPQ-SF** | McGill Pain Questionnaire - Short Form  SF-MPQ* | Melzack (1987). DOI: 10.1016/0304-3959(87)91074-8‌ | To measure clinical pain | 16 | 0 | Likert/VAS | Current perception | 2 | 0 | 0 | 0 |
| **NPSI** | Neuropathic Pain Symptom Inventory | Bouhassiraet al. (2004). DOI: 10.1016/j.pain.2003.12.024‌ | To evaluate the different symptoms of neuropathic pain | 12 | 0 | Likert | 24 hours | 0 | 0 | 1 | 0 |
| **PCS** | Pain Catastrophizing Scale | Sullivan et al. (1995). DOI: 10.1037//1040-3590.7.4.524  ‌ | To assess an individual's thoughts and feelings during experience of pain | 13 | 0 | Likert | Current perception | 0 | 0 | 1 | 1 |
| **PGI-C** | Patient Global Impression of Change  PGIC* | Guy (1976). | To assess patient perceptions of change in their condition | 1 | 0 | Likert | Since and for the duration of treatment | 12 | 1 | 0 | 2 |
| **PGI-I** | Patient Global Impression of Improvement  PGII* | Guy (1976). | To assess patient perceptions of improvement in their condition | 1 | 0 | Likert | In comparison to pre-treatment | 150 | 157 | 14 | 20 |
| **PGI-S** | Patient Global Impression of Severity  PGIS* | Guy (1976). | To determine the severity of a particular condition | 1 | 0 | Likert | 1 week | 3 | 32 | 1 | 3 |
| **PILL** | Pennebaker Inventory of Limbic Languidness | Pennebaker (1982). DOI: 10.1037/t05558-000  ‌ | To assess the frequency of common physical symptoms and sensations | 54 | 0 | Likert | Not specified | 0 | 0 | 1 | 0 |
| **SIP** | Sickness Impact Profile | Gilson et al. (1975). DOI: 10.2105/ajph.65.12.1304 | To assess changes in a person's behaviour due to sickness | 136 | 0 | Nominal | Current perception | 0 | 2 | 0 | 0 |
| **WHOQoL-BREF** | World Health Organization Quality of Life Assessment Instrument | THE WHOQOL GROUP. (1998). DOI: 10.1017/s0033291798006667  ‌ | To assess individuals' perceptions of the quality of their life | 26 | 0 | Likert | 2 weeks | 2 | 0 | 0 | 0 |
| **Wong Baker Pain Scale** | Wong Baker Pain Scale  Faces Pain Scale* | Wong & Baker (1988).  ‌ | To measure intensity of pain in children | 1 | 0 | Image scale | Current perception | 4 | 4 | 0 | 0 |

* Alternative terms or abbreviations for instrument

** Response categories - Likert: categorical/continuous data; NRS: numerical rating scale, continuous data; Dichotomous: categorical data, Yes/No responses; Nominal: categorical data, 3+ response options; VAS: visual analogue scale, continuous data; Free text: textual data
