# Supplementary material for: Patient‐Reported Outcome Measures Used to Assess Surgical Interventions for Pelvic Organ Prolapse, Stress Urinary Incontinence and Mesh Complications: A Scoping Review for the Development of the APPRAISE PROM
Source: BJOG. 2025 Sep 24;133(2):218–27. doi: 10.1111/1471-0528.18355 (PMC12678042; doi:10.1111/1471-0528.18355)
Supplement: Supplementary file 12 — Table S3: Table of condition‐specific PROMs—extracted data. [file BJO-133-218-s007.docx]

**Table S3: Condition-Specific PROMs- Extracted Data**

| **PROM (short title)** | **PROM**  **(long title)** | **Condition** | **Study reporting psychometric properties** | **PROM Aim** | **No. Core items** | **No. Bother items** | **Type of Response Categories**** | **Recall Period** | **No. POP Studies** | **No. SUI Studies** | **No. POP/SUI Combined Studies** | **No. Mesh Studies** |
| --- | --- | --- | --- | --- | --- | --- | --- | --- | --- | --- | --- | --- |
| **ADL** | Activities of Daily Living  Katz’s Index of Independence in Activities of Daily Living; KATZ ADL* | Chronic disease | Katz et al. (1963). DOI: [10.1001/jama.1963.03060120024016](https://doi.org/10.1001/jama.1963.03060120024016) | To measure ability to perform activities of daily living independently | 6 | 0 | Dichotomous | Not specified | 1 | 2 | 0 | 0 |
| **ALDS** | Academic Medical Centre Linear Disability Score | Chronic disease | Holman et al. (2005). DOI: [10.1186/1477-7525-3-83](https://doi.org/10.1186/1477-7525-3-83) | To measure the functional status of patients with a broad range of stable, chronic diseases | 77 | 0 | Dichotomous | Not specified | 0 | 1 | 0 | 0 |
| **APFQ** | Australian Pelvic Floor Questionnaire  Queensland Female Pelvic Floor Questionnaire - see also GFPFQ & GPOP-Q* | Pelvic floor disorders | Baessler et al. (2009). DOI: 10.1007/s00192-009-0997-4 | To assess pelvic floor function | 43 | 4 | Likert, nominal | 1 month | 7 | 0 | 0 | 0 |
| **BBUSQ-22** | Birmingham Bowel and Urinary Symptoms Questionnaire | Bowel and urinary dysfunction | Hiller et al. (2007). DOI: [10.1007/s00192-007-0308-x](https://doi.org/10.1007/s00192-007-0308-x) | To assess symptoms of bowel and urinary dysfunction in women | 22 | 0 | Likert | Not specified | 4 | 7 | 0 | 0 |
| **BFLUTS** | Bristol Female Lower Urinary Tract Symptoms  BFLUTS Short Form; BFLUTS-SF; sBFLUTS. This is now ICIQ-FLUTS* | Urinary incontinence | Brookes et al. (2004). DOI: [10.1016/j.ajog.2003.12.027](https://doi.org/10.1016/j.ajog.2003.12.027) | To assess symptoms of urinary incontinence in women | 19 | 0 | Likert | 1 month | 4 | 21 | 2 | 1 |
| **BRIQ** | Behavioural Response to Illness Questionnaire | Acute illness | Spence et al. (2005). DOI: [10.1017/s0033291704003484](https://doi.org/10.1017/s0033291704003484) | To assess behavioural responses in the development of ongoing medically unexplained syndromes | 21 | 0 | Likert | Not specified | 1 | 0 | 0 | 0 |
| **CCCS** | Cleveland Clinic Constipation Scoring System  Wexner Score for Constipation / Wexner-Agachan Constipation Scoring System/CCCS* | Constipation | Agachan et al. (1996). DOI: [10.1007/bf02056950](https://doi.org/10.1007/bf02056950) | To ascertain the severity of constipation in patients | 8 | 0 | Likert, nominal | Not specified | 40 | 0 | 0 | 0 |
| **CCI** | Charlson Co-Morbidity Index | Co-morbidities | Charlson et al. (1987). DOI: [10.1016/0021-9681(87)90171-8](https://doi.org/10.1016/0021-9681(87)90171-8) | To predict 10-year survival in patients with multiple comorbidities | 17 | 0 | Likert, nominal, dichotomous | Not specified | 2 | 1 | 0 | 0 |
| **CCIS** | Cleveland Clinic Incontinence Scoring System  Wexner Score for Constipation / Wexner-Agachan Constipation Scoring System/CCCS* | Faecal Incontinence | Jorge & Wexner (1993). DOI: [10.1007/BF02050307](https://doi.org/10.1007/BF02050307) | To assess the frequency and severity of faecal incontinence | 5 | 0 | Likert | Not specified | 48 | 0 | 0 | 0 |
| **CIRS** | Culminative Illness Rating Scale | Co-morbidities | Miller et al. (1992). DOI: [0.1016/0165-1781(92)90005-n](https://doi.org/10.1016/0165-1781(92)90005-n) | To measure the medical and psychiatric impairment of older adults | 14 | 0 | Nominal | Not specified | 1 | 0 | 0 | 0 |
| **CLSS** | Core Lower Urinary Tract Symptom Score | Lower urinary tract symptoms | Homma et al (2008). DOI: 10.1111/j.1442-2042.2008.02121.x | To assess core lower urinary tract symptoms | 10 | 3 | Likert, NRS | 1 week | 1 | 0 | 0 | 0 |
| **CRADI** | Colorectal Anal Distress Inventory  Subscale of PFDI* | Pelvic floor dysfunction / urogenital conditions | Barber et al. (2001). DOI: [10.1067/mob.2001.118659](https://doi.org/10.1067/mob.2001.118659) | To assess pelvic floor symptoms and bother, in particular colorectal-anal distress | 17 | 0 | Likert, dichotomous | 3 months | 7 | 0 | 0 | 0 |
| **CRADI-8** | Colorectal Anal Distress Inventory - 8 Items  Subscale of PFDI-20* | Pelvic floor dysfunction / urogenital conditions | Barber et al (2005). DOI: 10.1016/j.ajog.2004.12.025 | To assess pelvic floor symptoms and bother, in particular colorectal-anal distress | 8 | 0 | Likert, dichotomous | 3 months | 13 | 0 | 0 | 0 |
| **CRAIQ** | Colorectal-Anal Impact Questionnaire  Subscale of PFIQ* | Pelvic floor dysfunction / urogenital conditions | Barber et al. (2001). DOI: [10.1067/mob.2001.118659](https://doi.org/10.1067/mob.2001.118659) | To assess life impact in women with pelvic floor disorders | 31 | 0 | Likert | 3 months | 2 | 0 | 0 | 0 |
| **CRAIQ-7** | Colorectal-Anal Impact Questionnaire - 7 Items  Subscale of PFIQ-7* | Pelvic floor dysfunction / urogenital conditions | Barber et al. (2005). DOI: [10.1016/j.ajog.2004.12.025](https://doi.org/10.1016/j.ajog.2004.12.025) | To assess life impact in women with pelvic floor disorders | 7 | 0 | Likert | 3 months | 5 | 1 | 0 | 0 |
| **CSI** | Central Sensitisation Inventory | Musculo-skeletal pain | Mayer et al. (2012) DOI: 10.1111/j.1533-2500.2011.00493.x | To evaluate central sensitisation-related clinical symptoms | 33 | 0 | Likert, dichotomous | Not specified | 1 | 0 | 0 | 0 |
| **DIS** | Detrusor Instability Score | Urinary incontinence | Kauppila et al. (1982) DOI: 10.3109/00016348209156544 | To detect urge incontinence/SUI based on patient histories | 10 | 0 | Likert, dichotomous | Not specified | 1 | 10 | 0 | 0 |
| **ePAQ-PF** | Electronic Personal Assessment Questionnaire-Pelvic Floor | Pelvic floor dysfunction / urogenital conditions | Jones et al. (2008). DOI: 10.1007/s00192-008-0655-2 | To assess symptoms & impact to quality of life in women with pelvic floor disorders | 156 | 87 | Likert, free text | 4 weeks | 6 | 3 | 1 | 1 |
| **EPIQ** | Epidemiology of Prolapse and Incontinence Questionnaire | Pelvic floor dysfunction / urogenital conditions | Lukacz et al. (2005). DOI: [10.1007/s00192-005-1314-5](https://doi.org/10.1007/s00192-005-1314-5) | To screen for female pelvic floor disorders | 49 | 15 | Likert, VAS, dichotomous, nominal | Average day | 3 | 1 | 0 | 0 |
| **FIQL** | Faecal Incontinence Quality of Life Scale  American Society of Colon and Rectal Surgery Faecal Incontinence Quality of Life Questionnaire; FIQOL* | Faecal incontinence | Rockwood et al. (2000). DOI: [10.1007/bf02237236](https://doi.org/10.1007/bf02237236) | To measure the impact of treatment on faecal incontinence | 29 | 0 | Likert | 1 month | 8 | 0 | 0 | 0 |
| **FISI** | Faecal Incontinence Severity Index | Faecal incontinence | Rockwood et al. (1999). DOI: [10.1007/bf02236199](https://doi.org/10.1007/bf02236199) | To assess the severity of faecal incontinence | 4 | 0 | Likert | 1 month | 17 | 0 | 0 | 0 |
| **GIQLI** | Gastro-intestinal Quality of Life Index  GIQOL* | Gastro-intestinal diseases | Eypasch et al. (1995). DOI: [10.1002/bjs.1800820229](https://doi.org/10.1002/bjs.1800820229) | To assess quality of life specific to the gastrointestinal tract | 36 | 0 | Likert | 2 weeks | 6 | 0 | 0 | 0 |
| **ICIQ-B** | International Consultation on Incontinence Questionnaire - Anal Incontinence Symptoms and QoL module | Faecal incontinence | Cotterill et al. (2011) DOI: 10.1097/DCR.0b013e3182272128 | To evaluate symptoms of anal incontinence and impact on quality of life | 20 | 0 | Likert, NRS, free text | 3 months | 3 | 0 | 0 | 0 |
| **ICIQ-FLUTS** | International Consultation on Incontinence Questionnaire - Female Lower Urinary Tract Symptoms Modules  Derived from BFLUTS* | Lower urinary tract symptoms | Brookes et al. (2004). DOI: 10.1016/j.ajog.2003.12.027 | To assess female lower urinary tract symptom severity, impact on quality of life and treatment outcomes | 12 | 12 | Likert, NRS | 4 weeks | 2 | 24 | 3 | 1 |
| **ICIQ-FLUTSsex** | International Consultation on Incontinence Questionnaire - Female Sexual Matters Associated with Lower Urinary Tract Symptoms  Derived from BFLUTS* | Female sexual matters associated with lower urinary tract symptoms | Jackson et al. (1996). DOI: 10.1046/j.1464-410x.1996.00186.x | To evaluate sexual matters associated with female lower urinary tract symptoms | 4 | 4 | Likert, NRS | 4 weeks | 0 | 1 | 0 | 0 |
| **ICIQ-LUTSqol** | International Consultation on Incontinence Questionnaire -Lower Urinary Tract Symptoms Quality of Life Module  Derived from KHQ* | Lower urinary tract symptoms | Kelleher et al. (1997). DOI: 10.1111/j.1471-0528.1997.tb11006.x | To assess the impact of urinary incontinence on quality of life (social effects) | 20 | 19 | Likert, NRS | 4 weeks | 1 | 5 | 2 | 0 |
| **ICIQ-OAB** | International Consultation on Incontinence Questionnaire - Overactive Bladder Module  Derived from BFLUTS & ICSmale* | Overactive bladder symptoms | Jackson et al. (1996). DOI: 10.1046/j.1464-410x.1996.00186.x | To evaluate overactive bladder and related impact on quality of life | 4 | 4 | Likert, NRS | 4 weeks | 2 | 5 | 0 | 0 |
| **ICIQ-UI-SF** | International Consultation on Incontinence Questionnaire - Short Form  ICIQ; ICIQ-SF; ICIQ-UI* | Urinary incontinence | Avery et al. (2004). DOI: 10.1002/nau.20041 | To assess the symptoms and impact of urinary incontinence | 4 | 0 | Likert, NRS | 4 weeks | 60 | 181 | 15 | 3 |
| **ICIQ-VS** | International Consultation on Incontinence Questionnaire - Vaginal symptoms | Vaginal symptoms | Price et al. (2006). DOI: [10.1111/j.1471-0528.2006.00938.x](https://doi.org/10.1111/j.1471-0528.2006.00938.x) | To assess the severity and impact of vaginal symptoms and related sexual matters | 14 | 11 | Likert, VAS | 4 weeks | 4 | 1 | 4 | 1 |
| **IIQ** | Incontinence Impact Questionnaire  IIQ-30* | Urinary incontinence | Shumaker et al. (1994). DOI: 10.1007/bf00451721.   ‌ | To measure the impact of urinary incontinence on activities, roles, and emotional states in women | 30 | 0 | Likert | 1 month | 25 | 40 | 2 | 0 |
| **IIQ-7** | Incontinence Impact Questionnaire (short form - 7 items)  IIQ-SF; SIIQ* | Urinary incontinence | Uebersax et al. (1995). DOI: [10.1002/nau.1930140206](https://doi.org/10.1002/nau.1930140206) | To measure the impact of urinary incontinence on activities, roles, and emotional states in women | 7 | 0 | Likert | 1 month | 68 | 194 | 22 | 7 |
| **IPSS** | International Prostate Symptom Score  AUASS; AUASI; W-IPSS; IPSS-QOL* | Urinary symptoms | Barry et al. (1992). DOI: 10.1016/s0022-5347(17)36966-5 | To determine how bothersome urinary symptoms are and to assess effectiveness of treatment | 8 | 0 | Likert | 1 month | 9 | 12 | 1 | 3 |
| **I-QOL** | Urinary Incontinence-Specific Quality of Life  IQOL* | Urinary incontinence | Schurch et al. (2007). DOI: 10.1016/j.apmr.2007.02.009.   ‌ | To assess impact of urinary incontinence on quality of life | 22 | 0 | Likert | Not specified | 11 | 54 | 1 | 3 |
| **ISI** | Incontinence Severity Index  SIUIW; Severity Index for Urinary Incontinence in Women; Hunskarr Severity Index; Sandvik Severity Index* | Urinary incontinence | Sandvik et al. (2006). DOI: 10.1007/s00192-005-0060-z. | To assess severity of urinary incontinence | 2 | 0 | Likert | Not specified | 13 | 32 | 5 | 3 |
| **ISSI** | The Incontinence Symptom Severity Index | Urinary incontinence | Twiss et al. (2009). DOI: 10.1016/j.juro.2009.07.025 | To assess severity of urinary incontinence | 8 | 0 | Likert | 1 month | 0 | 1 | 0 | 1 |
| **KESS** | Knowles – Eccersley – Scott – Symptom | Constipation | Knowles et al. (2000). DOI: 10.1007/BF02236639 | To assist in the diagnosis of constipation | 11 | 0 | Likert, nominal | Not specified | 6 | 0 | 0 | 0 |
| **KHQ** | Kings Health Questionnaire  Kings Continence Questionnaire; KCQ; This is now ICIQ-LUTSqol* | Urinary incontinence | Kelleher et al. (1997). DOI: 10.1111/j.1471-0528.1997.  tb11006.x | To assess the impact of lower urinary tract symptoms on HRQL | 8 | 9 | Likert | Current perception | 8 | 72 | 5 | 3 |
| **MESA** | Medical, Epidemiologic, and Social Aspects of Aging Urinary Incontinence Questionnaire | Urinary incontinence | Diokno at al. (1986). DOI: 10.1016/s0022-5347(17)45  194-9. | To assess current urinary symptoms and history of urinary tract infections | 15 | 0 | Likert | Not specified | 7 | 33 | 3 | 1 |
| **M-ISI** | Michigan Incontinence Symptom Index | Urinary incontinence | Suskind et al. (2014). DOI: 10.1002/nau.22468 | To assess severity and bother related to urinary incontinence | 8 | 2 | Likert | 1 month | 0 | 1 | 0 | 1 |
| **OAB-q** | Overactive Bladder Questionnaire | Overactive bladder | Coyne et al. (2002). DOI: 10.1023/a:1016370925601. | To evaluate both continent and incontinent symptoms of OAB and their impact on HRQL | 33 | 33 | Likert | 4 weeks | 2 | 0 | 0 | 0 |
| **OAB-q-SF** | Overactive Bladder Questionnaire - Short Form | Overactive bladder | Coyne et al. (2015). DOI: 10.1002/nau.22559 | To evaluate both continent and incontinent symptoms of OAB and their impact on HRQL | 8 | 6 | Likert, dichotomous | 4 weeks | 2 | 1 | 0 | 0 |
| **OABSS** | Overactive Bladder Symptom Score | Overactive bladder | Homma et al. (2006) DOI: 10.1016/j.urology.2006.02.042 | To measure overactive bladder symptoms | 4 | 0 | Likert | 1 week | 9 | 6 | 1 | 0 |
| **OAB-V8** | Overactive Bladder Questionnaire | Overactive bladder | Coyne et al. (2005). DOI. 10.1007/BF02850085 | To identify patients with bothersome overactive bladder symptoms | 8 | 0 | Likert | Not specified | 0 | 3 | 1 | 0 |
| **ODS** | Obstructed Defecation Syndrome Score  Longo Score; ODS-S* | Obstructive defecation | Altomare et al. (2008). DOI: 10.1111/  j.1463-1318.  2007.01262.x | To quantify severity of obstructed defaecation syndrome | 5 | 0 | Likert | Not specified | 18 | 0 | 0 | 0 |
| **PAC-QOL** | Patient Assessment of Constipation Quality of Life | Constipation | Marquis et al. (2005). DOI: 10.1080/00365520510012208 | To evaluate constipation over time | 28 | 0 | Likert | 2 weeks | 9 | 0 | 0 | 0 |
| **PAC-SYM** | Patient Assessment of Constipation Symptoms | Constipation | Frank et al. (1999). DOI: 10.1080/003655299750025327. | To evaluate severity of constipation | 12 | 0 | Likert | 2 weeks | 3 | 0 | 0 | 0 |
| **PFBQ** | Pelvic Floor Bother Questionnaire | Pelvic floor dysfunction / Urogenital conditions | Peterson et al. (2010). DOI: 10.1007/s00192-010-1148-7 | To assess presence of common pelvic floor disorders and related bother | 9 | 8 | Likert | 1 month | 0 | 0 | 1 | 0 |
| **PFDI** | Pelvic Floor Distress Inventory (46 item)  PFDI-46* | Pelvic floor dysfunction / Urogenital conditions | Barber et al. (2001). DOI: 10.1067/mob.2001.118659 | To provide a symptom inventory and to measure the degree of bother and distress caused by the broad array of pelvic floor symptoms | 46 | 46 | Likert, dichotomous | 3 months | 51 | 5 | 6 | 2 |
| **PFDI-20** | Pelvic Floor Distress Inventory Questionnaire (20 item) | Pelvic floor dysfunction / Urogenital conditions | Barber et al. (2005). DOI: 10.1016/j.ajog.2004.12.025 | To provide a symptom inventory and to measure the degree of bother and distress caused by the broad array of pelvic floor symptoms | 20 | 20 | Likert, dichotomous | 3 months | 246 | 13 | 9 | 8 |
| **PFIQ** | Pelvic Floor Impact Questionnaire  PFIQ-31* | Pelvic floor dysfunction / Urogenital conditions | Barber et al.(2001). DOI: 10.1067/mob.2001.118659 | To assess life impact in patients with female pelvic floor disorders | 93 | 0 | Likert | 3 months | 29 | 3 | 5 | 1 |
| **PFIQ-7** | Pelvic Floor Impact Questionnaire - Short Form (7 item) | Pelvic floor dysfunction / Urogenital conditions | Barber et al. (2005). DOI: 10.1016/j.ajog.2004.12.025 | To assess life impact in patients with female pelvic floor disorders | 21 | 0 | Likert | 3 months | 136 | 15 | 5 | 5 |
| **PISQ-12** | Pelvic Organ Prolapse Urinary Incontinence Sexual Questionnaire-Short Form (12 items) | Pelvic floor disorders | Rogers et al. (2003). DOI: 10.1007/s00192-003-1063-2 | To evaluate sexual function in patients with female pelvic floor disorders | 12 | 0 | Likert | 6 months | 190 | 70 | 12 | 3 |
| **PISQ-31** | Pelvic Organ Prolapse Urinary Incontinence Sexual Questionnaire  PISQ* | Pelvic floor disorders | Rogers et al. (2001). DOI: 10.1067/mob.2001.111100 | To evaluate sexual function in patients with female pelvic floor disorders | 31 | 0 | Likert | 6 months | 11 | 2 | 2 | 0 |
| **PISQ-IR** | Pelvic Organ Prolapse/Urinary Incontinence Sexual Questionnaire - IUGA Revised. | Pelvic floor disorders | Rogers et al. (2013). DOI: 10.1007/s00192-012-2020-8 | To evaluate sexual function in patients with female pelvic floor disorders | 20 | 0 | Likert | Current perception | 23 | 4 | 0 | 0 |
| **POPDI** | Pelvic Organ Prolapse Distress Inventory  Subscale of PFDI* | Pelvic organ prolapse | Barber et al. (2001). DOI: 10.1067/mob.2001.118659 | To assess symptom distress in patients with female pelvic floor disorders (especially pelvic organ prolapse) | 16 | 16 | Likert, dichotomous | 3 months | 12 | 0 | 0 | 0 |
| **POPDI-6** | Pelvic Organ Prolapse Distress Inventory - Short Form (6 items)  Subscale of PFDI-20* | Pelvic organ prolapse | Barber et al. (2005). DOI: 10.1016/j.ajog.2004.12.025 | To assess symptom distress in patients with female pelvic floor disorders (especially pelvic organ prolapse) | 6 | 6 | Likert, dichotomous | 3 months | 41 | 1 | 3 | 0 |
| **POPIQ** | Pelvic Organ Prolapse Impact Questionnaire  Subscale of PFIQ* | Pelvic organ prolapse | Barber et al. (2001). DOI: 10.1067/mob.2001.118659 | To measure the impact of  vaginal/prolapse symptoms on HRQL | 31 | 0 | Likert | 3 months | 2 | 0 | 0 | 0 |
| **POPIQ-7** | Pelvic Organ Prolapse Impact Questionnaire - Short Form (7 items)  Subscale of PFIQ-7* | Pelvic organ prolapse | Barber et al. (2005). DOI: 10.1016/j.ajog.2004.12.025 | To measure the impact of  vaginal/prolapse symptoms on HRQL | 7 | 0 | Likert | 3 months | 5 | 0 | 0 | 0 |
| **POP-SS** | Pelvic Organ Prolapse Symptom Score | Pelvic organ prolapse | Hagen et al. (2009). DOI: 10.1111/j.1471-0528.  2008.01903.x | To measure symptoms of pelvic organ prolapse | 7 | 1 | Likert | 4 weeks | 9 | 0 | 0 | 0 |
| **PPBC** | Patient Perception of Bladder Condition | Overactive bladder | Coyne et al. (2006). DOI: 10.1016/j.eururo.2006.01.007 | To assess bladder and urinary symptoms | 1 | 0 | Likert | Current perception | 1 | 0 | 1 | 0 |
| **PPIUS** | Patient Perception of Intensity of Urgency Scale  Patient Perception of Urgency Severity; PPUS* | Urinary incontinence | Notte et al. (2012). DOI: 10.1186/1471-2490-12-26 | To measure the intensity of urgency associated with each urinary or incontinence episode | 2 | 0 | Likert | Each urinary or UI episode | 0 | 8 | 0 | 1 |
| **P-QOL** | Pelvic Organ Prolapse Quality of Life | Pelvic organ prolapse | Digesu et al. (2005). DOI: 10.1007/s00192-004-1225-x | To assess the impact of female pelvic organ prolapse on HRQL | 38 | 0 | Likert | Current perception | 52 | 2 | 8 | 0 |
| **RUIS** | Revised Urinary Incontinence Scale | Urinary incontinence | Sansoni et al. (2012) [www.ics.org/Abstracts/Publish/134/000370.pdf](https://www.ics.org/Abstracts/Publish/134/000370.pdf) | To measure urinary incontinence and outcomes following treatment | 5 | 0 | Likert | Current perception | 0 | 2 | 0 | 0 |
| **SEAPI-QMM** | SEAPI-QMM Quality of Life Index  SEAPI-QOL (QoL index only)* | Urinary incontinence | Raz & Erickson. (1992). DOI: 10.1002/nau.1930110302‌ | To assess urinary incontinence and provide a classification system for symptoms | 8 | 0 | Likert | Current perception | 4 | 15 | 1 | 1 |
| **SII** | Symptom Impact Index | Urinary incontinence | Black et al. (1996). DOI: 10.1002/(sici)1520-6777(1996)15:6%3C630::aid-nau4%3E3.0.co;2-g. | To measure female stress incontinence severity and the impact of symptom bother | 4 | 0 | Likert, dichotomous | Not specified | 0 | 2 | 0 | 0 |
| **SPS-Q** | Sheffield Prolapse Symptoms Questionnaire  SPSQ* | Pelvic organ prolapse | Bradshaw et al. (2006). DOI: 10.1080/01443610500537989 | To quantify symptoms relating to pelvic organ prolapse | 25 | 25 | Likert | 1 month | 1 | 0 | 0 | 0 |
| **SSI** | Symptom Severity Index | Urinary incontinence | Black et al. (1996). DOI: 10.1002/(sici)1520-6777(1996)15:6%3C630::aid-nau4%3E3.0.co;2-g. | To measure female stress incontinence severity as well as the impact of symptom bother | 5 | 0 | Likert, dichotomous | Not specified | 0 | 7 | 1 | 0 |
| **St Marks** | St Marks Incontinence Score  Vaizey Incontinence Score* | Faecal incontinence | Paka et al. (2016). DOI: 10.1007/s10151-015-1397-z | To assess severity of anal incontinence | 8 | 0 | Likert, dichotomous | Not specified | 4 | 0 | 0 | 0 |
| **SUIQQ** | Stress and Urge Incontinence and Quality of Life Questionnaire | Urinary incontinence | Kulseng-Hanssen & Borstad. (2003). DOI: 10.1111/j.1471-0528.2003.01406.x | To measure the severity of urinary incontinence symptoms and HRQL in women | 12 | 1 | Likert, dichotomous | Not specified | 0 | 4 | 0 | 1 |
| **TBS** | Treatment Benefit Scale | Overactive bladder | Colman et al. (2008). DOI: 10.1016/j.urology.2008.05.033 | To assess subjective outcomes in the treatment of overactive bladder | 1 | 0 | Likert | Since start of treatment | 0 | 1 | 0 | 0 |
| **UDI** | Urogenital Distress Inventory  Subscale of PFDI* | Pelvic floor dysfunction /Urogenital conditions | Shumaker, et al. (1994). DOI: 10.1007/bf00451721   ‌ | To measure the impact of urinary incontinence on activities, roles, and emotional states in women | 28 | 0 | Likert | 3 months | 38 | 55 | 2 | 2 |
| **UDI-6** | Urogenital Distress Inventory-Short Form (6 items)  Subscale of PFDI-20* | Pelvic floor dysfunction /Urogenital conditions | Uebersax et al. (1995). DOI: 10.1002/nau.1930140206 | To measure the impact of urinary incontinence on activities, roles, and emotional states in women | 6 | 0 | Likert | 3 months | 104 | 215 | 27 | 19 |
| **UIQ** | Urinary Impact Questionnaire  Subscale of PFIQ* | Pelvic floor dysfunction / Urogenital conditions | Barber et al. (2001). DOI: 10.1067/mob.2001.118659 | To measure the impact of bladder, bowel, and vaginal symptoms on a woman’s daily activities, relationships and emotions | 31 | 0 | Likert | 3 months | 3 | 0 | 1 | 0 |
| **UIQ-7** | Urinary Impact Questionnaire - Short Form (7 items)  Subscale of PFIQ-7* | Pelvic floor dysfunction / Urogenital conditions | Barber et al. (2005).DOI: 10.1016/j.ajog.2004.12.025   ‌ | To measure the impact of bladder, bowel, and vaginal symptoms on a woman’s daily activities, relationships and emotions | 7 | 0 | Likert | 3 months | 4 | 2 | 0 | 1 |
| **UISS** | Urinary Incontinence Severity Score | Urinary incontinence | Stach-Lempinen et al. (2001). DOI: 10.1080/003655901753367587 | To measure female urinary incontinence and impact on HRQL (incl. social interactions, physical activities and sexual function) | 10 | 0 | Likert | Not specified | 1 | 13 | 0 | 0 |
| **ULCQ** | Urinary Leakage Circumstances Questionnaire | Urinary incontinence | Nyangoh Timoh et al. (2017). DOI: 10.1016/j.urology.2017.05.009. | To assess female urinary leakage circumstances | 25 | 0 | Likert, Free text | Not specified | 0 | 1 | 0 | 0 |
| **UPS** | Urgency Perception Scale | Overactive bladder | Cardozo et al. (2005). DOI: 10.1111/j.1464-410X.2005.  05345.x. | To assess urinary urgency in patients with overactive bladder syndrome | 1 | 0 | Nominal | Not specified | 1 | 8 | 0 | 0 |
| **USP** | Urinary Symptoms Profile | Urinary incontinence | Haab et al. (2008). DOI: 10.1016/j.urology.2007.11.100 | To assess stress, urge or overactive bladder urinary symptoms or urinary obstructive symptoms | 13 | 0 | Likert, NRS | 4 weeks | 1 | 2 | 0 | 1 |
| **USS** | Urinary Sensation Scale | Urinary incontinence | Coyne et al. (2011). DOI: 10.1002/nau.21005 | To assess feelings of urinary urgency associated with each urination | 1 | 0 | Likert | Not specified | 0 | 2 | 0 | 0 |

* Alternative terms or abbreviations for instrument

** Response categories - Likert: categorical/continuous data; NRS: numerical rating scale, continuous data; Dichotomous: categorical data, Yes/No responses; Nominal: categorical data, 3+ response options; VAS: visual analogue scale, continuous data; Free text: textual data
