# Supplementary material for: Patient‐Reported Outcome Measures Used to Assess Surgical Interventions for Pelvic Organ Prolapse, Stress Urinary Incontinence and Mesh Complications: A Scoping Review for the Development of the APPRAISE PROM
Source: BJOG. 2025 Sep 24;133(2):218–27. doi: 10.1111/1471-0528.18355 (PMC12678042; doi:10.1111/1471-0528.18355)
Supplement: Supplementary file 13 — Table S4: Table of preference‐based PROMs—extracted data. [file BJO-133-218-s020.docx]

**Table S4: Preference-Based PROMs – Extracted Data**

| **PROM (short title)** | **PROM (long title)** | **Study reporting psychometric properties** | **PROM Aim** | **No. Core items** | **No. Bother items** | **Type of Response Categories**** | **Recall Period** | **No. POP Studies** | **No. SUI Studies** | **No. POP/SUI Combined Studies** | **No. Mesh Studies** |
| --- | --- | --- | --- | --- | --- | --- | --- | --- | --- | --- | --- |
| 15D | 15-Dimensional health-related quality of life measure | Sintonen. (2017). DOI: 10.4225/03/59389bbded953   ‌ | To measure HRQL and its utility, and to evaluate the efficacy/ effectiveness of health interventions | 15 | 0 | Nominal | Current perception | 7 | 2 | 0 | 0 |
| EQ-5D (Unspecified version) | Euro QOL 5D (Unspecified version) | EuroQol Group. (1990). DOI: 10.1016/0168-8510(90)90421-9   ‌ | To assess health outcome from a wide variety of interventions, for the purposes of evaluation, allocation and monitoring | 6 | 0 | Likert/ VAS | Current perception | 18 | 18 | 1 | 1 |
| EQ-5D-3L | Euro QOL 5D - 3 Level version | EuroQol Group. (1990). DOI: 10.1016/0168-8510(90)90421-9   ‌ | To assess health outcome from a wide variety of interventions, for the purposes of evaluation, allocation and monitoring | 6 | 0 | Likert/ VAS | Current perception | 5 | 3 | 0 | 0 |
| EQ-5D-5L | Euro QOL 5D - 5 Level version | Herdman et al. (2011). DOI: 10.1007/s11136-011-9903-x | To assess health outcome from a wide variety of interventions, for the purposes of evaluation, allocation and monitoring | 6 | 0 | Likert/ VAS | Current perception | 3 | 3 | 0 | 1 |
| EQ-VAS | Euro QOL Visual Analogue Scale | EuroQol Group. (1990). DOI: 10.1016/0168-8510(90)90421-9 | To assess patients’ self-rated health using a vertical visual analogue scale | 1 | 0 | VAS | Current perception | 1 | 0 | 0 | 1 |
| HUI | Health Utilities Index | Torrance et al. (1995). DOI: 10.2165/00019053-199507060-00005 | To describe health status, measure within-attribute morbidity and HRQL, and produce utility scores | 7 | 0 | Nominal | Not specified | 0 | 2 | 0 | 0 |
| iMCQ | Medical Consumption Questionnaire  iMTA MCQ* | iMTA Productivity and Health Research Group (2018). | To measure medical costs for use in economic evaluations in healthcare | 18 | 0 | Dichotomous, free-text, nominal | 3 months | 2 | 0 | 0 | 0 |
| iPCQ | Productivity Cost Questionnaire (iPCQ)  iMTA PCQ* | Bouwmans et al. (2015). DOI: 10.1016/j.jval.2015.05.009 | To measure and value productivity losses for use in economic evaluations in healthcare | 18 | 0 | Dichotomous, free-text, NRS | 4 weeks | 2 | 0 | 0 | 0 |
| PROMIS Pain Intensity SF 3A | Patient-Reported Outcomes Measurement Information System Pain Intensity Short Form | Broderick et al. (2013). DOI: 10.1002/acr.22025   ‌ | To assess the amount of pain experienced | 3 | 0 | Likert | 7 days | 1 | 0 | 0 | 0 |
| PROMIS-PI | Patient-Reported Outcomes Measurement Information System Pain Interference | Amtmann et al. (2010). DOI: 10.1016/j.pain.2010.04.025   ‌ | To assess the negative effects of pain on functioning | 6 | 0 | Likert | 7 days | 1 | 0 | 0 | 0 |
| PROMIS-SexFS | Patient-Reported Outcomes Measurement Information System Sexual Function and Satisfaction | Flynn et al. (2013). DOI: 10.1186/1477-7525-11-40 | To measure sexual function and satisfaction in female populations | 79 | 0 | Likert | 30 days | 1 | 0 | 0 | 0 |
| PROMIS (unspecified) | Patient-Reported Outcomes Measurement Information System (unspecified measure) | X | X | X | X | X | X | 2 | 2 | 0 | 0 |
| SF-12 | 12-Item Short Form Survey  RAND-12* | Ware et al. (1996). DOI: 10.1097/00005650-199603000-00003   ‌ | To measure generic health concepts relevant across age, disease, and treatment groups | 12 | 0 | Likert/  Dichotomous | 4 weeks | 12 | 9 | 1 | 2 |
| SF-36 | 36-Item Short Form Survey  RAND-36* | Ware et al. (1992). DOI: 10.1097/00005650-199206000-00002   ‌ | To measure generic health concepts relevant across age, disease, and treatment groups | 36 | 0 | Likert/  Dichotomous | 4 weeks | 33 | 16 | 6 | 0 |
| SWLS | Satisfaction with Life Scale | Diener et al. (1985). DOI: 10.1207/s15327752jpa4901_13   ‌ | To assess global life satisfaction | 5 | 0 | Likert | Not specified | 1 | 0 | 0 | 0 |

* Alternative terms or abbreviations for instrument

** Response categories - Likert: categorical/continuous data; NRS: numerical rating scale, continuous data; Dichotomous: categorical data, Yes/No responses; Nominal: categorical data, 3+ response options; VAS: visual analogue scale, continuous data; Free text: textual data
