# Supplementary material for: Patient‐Reported Outcome Measures Used to Assess Surgical Interventions for Pelvic Organ Prolapse, Stress Urinary Incontinence and Mesh Complications: A Scoping Review for the Development of the APPRAISE PROM
Source: BJOG. 2025 Sep 24;133(2):218–27. doi: 10.1111/1471-0528.18355 (PMC12678042; doi:10.1111/1471-0528.18355)
Supplement: Supplementary file 14 — Table S5: Table of surgery‐specific PROMs—extracted data. [file BJO-133-218-s016.docx]

**Table S5: Surgery-Specific PROMs – Extracted Data**

| **PROM (short title)** | **PROM**  **(long title)** | **Condition** | **Study reporting psychometric properties** | **PROM Aim** | **No. Core items** | **No. Bother items** | **Type of Response Categories**** | **Recall Period** | **No. POP Studies** | **No. SUI Studies** | **No. POP/SUI Combined Studies** | **No. Mesh Studies** |
| --- | --- | --- | --- | --- | --- | --- | --- | --- | --- | --- | --- | --- |
| AAS | Activities Assessment Scale | Pelvic floor dysfunction / Urogenital conditions   *Originally validated for male hernia surgery | Barber et al. (2012). DOI: 10.1097/spv.0b013e31825e6422. | To evaluate functional activity in women after surgery | 13 | 0 | Likert | 24 hours | 5 | 1 | 0 | 0 |
| CARE | Convalescence And Recovery Evaluation | Abdominal and pelvic surgery | Hollenbeck et al.(2008). DOI: 10.1007/s11136-008-9366-x‌ | To assess health status following abdominal and pelvic surgery | 20 | 7 | Likert | 7 days | 1 | 0 | 0 | 0 |
| DRS-PFD | Decision Regret Scale - Pelvic Floor Disorders | Pelvic floor dysfunction / Urogenital conditions | Sung et al. (2008). DOI: 10.1016/j.ajog.2007.12.035 | To measure regret in women following healthcare decisions about surgery for pelvic floor disorders | 5 | 0 | Likert | Current perception | 7 | 1 | 1 | 1 |
| GUTSS | Genito-Urinary Treatment Satisfaction Scale | Urinary incontinence | Hawthorne & Harmer (2017). DOI: 10.4225/03/5934f47f7f463 | To measure women’s satisfaction with treatment outcomes for SUI and other related disorders | 10 | 0 | Likert,  dichotomous | Since most recent surgery | 0 | 2 | 0 | 0 |
| ICS/IUGA | ICS/IUGA Complications Classification  IUGA/ICS* | Pelvic floor dysfunction / Urogenital conditions | Haylen et al. (2010). DOI: 10.1002/nau.21036   ‌ | To assess prosthesis and grafts complications in female pelvic floor surgery | 4 | 0 | Nominal | Not specified | 8 | 3 | 0 | 1 |
| PONV | Post Operative Nausea and Vomiting Scale | Post-operative nausea/vomiting | Myles & Wengritzky. (2012). DOI: 10.1093/bja/aer505.   ‌ | To assess nausea and vomiting perioperatively | 4 | 0 | Likert, nominal | 6 hours post-surgery or discharge | 2 | 0 | 0 | 0 |
| POSAS | Patient and Observer Scar Assessment Questionnaire | Scarring | van de Kar et al. (2005). DOI: 10.1097/01.prs.0000172982.43599.d6.‌ | To measure scar quality in all types of scars | 14 | 0 | Likert | Current perception | 1 | 0 | 0 | 0 |
| PSAQ | Patient Scar Assessment Questionnaire | Scarring | Durani et al. (2009). DOI: 10.1097/prs.0b013e3181a205de | To measure patient's perception of scarring | 39 | 0 | Likert | Current perception | 1 | 0 | 0 | 0 |
| PSR-13 | Postdischarge Surgical Recovery Scale - 13 Item | Generic | ‌Brandon et al (2022). DOI: 10.1007/s00192-022-05372-6.   ‌ | To assess post-operative quality of recovery | 13 | 0 | NRS | Current perception | 1 | 0 | 0 | 0 |
| PSR-15 | Postdischarge Surgical Recovery Scale - 15 Item | Generic | Kleinbeck (2000). DOI: 10.1002/1098-240x(200012)23:6%3C461::aid-nur5%3E3.0.co;2-s | To assess post-operative quality of recovery | 15 | 0 | NRS | Current perception | 2 | 0 | 0 | 0 |
| QoR-15 | Quality of Recovery - Short Form | Generic | Chazapis et al. (2016). DOI: 10.1093/bja/aev413 | To assess post-operative quality of recovery | 15 | 0 | NRS | 24 hours | 0 | 1 | 0 | 0 |
| QoR-40 | Quality of Recovery (40 Item) | Generic | Myles et al. (2000). DOI: 10.1093/oxfordjournals.bja.a013366.   ‌ | To assess post-operative quality of recovery | 40 | 0 | NRS | 24 hours | 1 | 0 | 0 | 0 |
| RI-10 | Recovery Index-10 | Pelvic floor dysfunction / Urogenital conditions | Kluivers et al. (2008). DOI: 10.1016/j.surg.2008.03.027.   ‌ | To assess post-operative quality of recovery | 10 | 0 | Likert | Not specified | 2 | 0 | 0 | 0 |
| SDS-PFD | Satisfaction with Decision Scale -Pelvic Floor Disorders | Pelvic floor dysfunction / Urogenital conditions | Sung et al. (2008). DOI: 10.1016/j.ajog.2007.12.035 | To assess patient satisfaction with pelvic floor surgical treatment | 6 | 0 | Likert | Not specified | 6 | 0 | 1 | 1 |
| SPS | Surgical Pain Scales | Pelvic floor dysfunction / Urogenital conditions   *Originally validated for male hernia surgery | Barber et al. (2012). DOI: 10.1097/spv.0b013e31825d65aa.   ‌ | To measure pain and discomfort in women undergoing surgery for POP or SUI | 4 | 0 | NRS | 24 hours | 4 | 0 | 0 | 0 |
| SSQ-8 | Surgical Satisfaction Questionnaire | Generic | Haff et al. (2011). DOI: 10.1016/j.jmig.2011.08.171.   ‌ | To assess patient satisfaction following surgery to correct prolapse/  incontinence | 8 | 0 | Likert | Since surgery | 7 | 0 | 2 | 1 |
| UIOS | Urinary Incontinence Outcome Score  Groutz Anti-Incontinence Surgery Response Score* | Urinary incontinence | Groutz et al.(2000). DOI:10.1002/(sici)1520-6777(2000)19:2<127::aid-nau3>3.0.co;2-k‌ | To evaluate urinary incontinence following surgical treatment | 16 | 0 | Likert, nominal, dichotomous, NRS | Current perception | 0 | 2 | 0 | 1 |

* Alternative terms or abbreviations for instrument

** Response categories - Likert: categorical/continuous data; NRS: numerical rating scale, continuous data; Dichotomous: categorical data, Yes/No responses; Nominal: categorical data, 3+ response options; VAS: visual analogue scale, continuous data; Free text: textual data
