# Supplementary material for: Patient‐Reported Outcome Measures Used to Assess Surgical Interventions for Pelvic Organ Prolapse, Stress Urinary Incontinence and Mesh Complications: A Scoping Review for the Development of the APPRAISE PROM
Source: BJOG. 2025 Sep 24;133(2):218–27. doi: 10.1111/1471-0528.18355 (PMC12678042; doi:10.1111/1471-0528.18355)
Supplement: Supplementary file 15 — Table S6: Table of sex‐specific PROMs—extracted data. [file BJO-133-218-s001.docx]

**Table S6: Sex-Specific PROMs – Extracted Data**

| **PROM (short title)** | **PROM (long title)** | **Study reporting psychometric properties** | **PROM Aim** | **No. Core items** | **No. Bother items** | **Type of Response Categories**** | **Recall Period** | **No. POP Studies** | **No. SUI Studies** | **No. POP/SUI Combined Studies** | **No. Mesh Studies** |
| --- | --- | --- | --- | --- | --- | --- | --- | --- | --- | --- | --- |
| BISF-W | Brief Index Sexual Functioning for Women | Taylor et al. (1994). DOI: 10.1007/bf01541816 | To assess current levels of female sexual functioning and satisfaction | 22 | 0 | Likert, nominal, dichotomous | 1 month | 1 | 0 | 0 | 0 |
| FSDS | Female Sexual Distress Scale | Derogatis et al. (2002). DOI: 10.1080/00926230290001448 | To measure sexually related personal distress in women | 12 | 0 | Likert | 30 days | 4 | 1 | 1 | 0 |
| FSDS-R | Female Sexual Distress Scale - Revised | Derogatis et al. (2008). DOI: 10.1111/j.1743-6109.2007.00672.x | To measure sexually related personal distress in women | 12 | 1 | Likert | 30 days | 2 | 0 | 0 | 0 |
| FSFI | Female Sexual Function Index | Rosen et al. (2000). DOI: 10.1080/009262300278597 | To measure female sexual arousal/functioning in women | 19 | 0 | Likert | 30 days | 45 | 40 | 11 | 6 |
| GRISS | Golombok Rust Inventory of Sexual Satisfaction (Female Scale) | Rust et al. (1986) DOI: 10.1007/BF01542223 | To assess sexual dysfunction in heterosexual couples (female scale) | 28 | 0 | Likert | Not specified | 1 | 0 | 2 | 0 |
| LSS | Libido Scoring System | Api et al. (2005) | To assess sexual function | 4 | 0 | Likert, nominal, dichotomous | Not specified | 0 | 1 | 0 | 0 |
| QS-F | Quality of Sexual Function Questionnaire  FSQ* | Heinemann et al. (2005). DOI: 10.1111/j.1743-6109.2005.20108.x. | To assess sexual function | 40 | 0 | Likert, nominal, free-text | 1 month | 1 | 0 | 0 | 0 |
| SSAQ | Subjective Sexual Arousal and Affect Questionnaire | Laan et al. (1995). DOI: 10.1111/j.1469-8986.1995.tb02099.x. | To measure sexual feelings and affect after erotic stimulus exposure | 37 | 0 | Likert | During the [type of erotic exposure] | 2 | 0 | 0 | 0 |

* Alternative terms or abbreviations for instrument

** Response categories - Likert: categorical/continuous data; NRS: numerical rating scale, continuous data; Dichotomous: categorical data, Yes/No responses; Nominal: categorical data, 3+ response options; VAS: visual analogue scale, continuous data; Free text: textual data
