# Supplementary material for: Patient‐Reported Outcome Measures Used to Assess Surgical Interventions for Pelvic Organ Prolapse, Stress Urinary Incontinence and Mesh Complications: A Scoping Review for the Development of the APPRAISE PROM
Source: BJOG. 2025 Sep 24;133(2):218–27. doi: 10.1111/1471-0528.18355 (PMC12678042; doi:10.1111/1471-0528.18355)
Supplement: Supplementary file 16 — Table S7: Table of mental health‐specific PROMs—extracted data. [file BJO-133-218-s002.docx]

**Table S7: Mental Health-Specific PROMs – Extracted Data**

| **PROM (short title)** | **PROM (long title)** | **Condition** | **Study reporting psychometric properties** | **PROM Aim** | **No. Core items** | **No. Bother items** | **Type of Response Categories**** | **Recall Period** | **No. POP Studies** | **No. SUI Studies** | **No. POP/SUI Combined Studies** | **No. Mesh Studies** |
| --- | --- | --- | --- | --- | --- | --- | --- | --- | --- | --- | --- | --- |
| BAI | Beck Anxiety Inventory | Anxiety disorders | Beck et al (1988). DOI: [10.1037/0022-006X.56.6.893](https://doi.org/10.1037/0022-006X.56.6.893) | To measure the severity of anxiety in psychiatric populations | 21 | 21 | Likert | 1 month | 1 | 0 | 1 | 0 |
| BDI | Beck Depression Inventory | Depression | Beck et al. (1996) | To measure the severity of depression in adults and adolescents | 21 | 0 | Likert | Not specified | 1 | 1 | 1 | 0 |
| BDI-SF | Beck Depression Inventory -Short Form | Depression | ‌Furlanetto,et al. (2005). [DOI: 10.1016/j.jad.2004.12.011](https://doi.org/10.1016/j.jad.2004.12.011) | To measure the severity of depression in adults and adolescents | 13 | 0 | Likert | Not specified | 1 | 0 | 0 | 0 |
| GAD-7 | Generalised Anxiety Disorder Scale | Anxiety disorders | Spitzer et al. (2006). DOI: [10.1001/archinte.166.10.1092](https://doi.org/10.1001/archinte.166.10.1092)   ‌ | To identify probable cases of Generalized Anxiety Disorder and assess symptom severity | 8 | 0 | Likert | 2 weeks | 1 | 0 | 0 | 0 |
| GDS-SF | Geriatric Depression Scale - Short Form | Anxiety and depression | Lesher & Berryhill (1994). DOI: [10.1002/1097-4679(199403)50:2%3C256::aid-jclp2270500218%3E3.0.co;2-e](https://doi.org/10.1002/1097-4679(199403)50:2%3C256::aid-jclp2270500218%3E3.0.co;2-e) | To screen for depression in frail community-dwelling older populations | 15 | 0 | Dichotomous | Not specified | 1 | 0 | 0 | 0 |
| HADS | Hospital Anxiety and Depression Scale | Anxiety and depression | Zigmond & Snaith (1983). | To detect states of anxiety and depression | 14 | 0 | Likert | Not specified | 0 | 1 | 1 | 0 |
| PHQ-9 | Patient Health Questionnaire- 9 Item | Depression | Kroenke et al. (2001) DOI: 10.1046/j.1525-1497.2001.016009606.x | To measure depression severity and to diagnose depressive disorders | 9 | 0 | Likert | 2 weeks | 6 | 3 | 0 | 0 |
| STAI | Spielberger State-Trait Anxiety Inventory | Anxiety disorders | Spielberger, et al (1983). | To measure anxiety in adults | 20 | 0 | Likert | Current perception | 0 | 1 | 0 | 0 |

* Alternative terms or abbreviations for instrument

** Response categories - Likert: categorical/continuous data; NRS: numerical rating scale, continuous data; Dichotomous: categorical data, Yes/No responses; Nominal: categorical data, 3+ response options; VAS: visual analogue scale, continuous data; Free text: textual data
