# Supplementary material for: Patient‐Reported Outcome Measures Used to Assess Surgical Interventions for Pelvic Organ Prolapse, Stress Urinary Incontinence and Mesh Complications: A Scoping Review for the Development of the APPRAISE PROM
Source: BJOG. 2025 Sep 24;133(2):218–27. doi: 10.1111/1471-0528.18355 (PMC12678042; doi:10.1111/1471-0528.18355)
Supplement: Supplementary file 17 — Table S8: Table of body image‐specific PROMs—extracted data. [file BJO-133-218-s010.docx]

**Table S8: Body Image-Specific PROMs – Extracted Data**

| **PROM (short title)** | **PROM (long title)** | **Condition** | **Study reporting psychometric properties** | **PROM Aim** | **No. Core items** | **No. Bother items** | **Type of Response Categories**** | **Recall Period** | **No. POP Studies** | **No. SUI Studies** | **No. POP/SUI Combined Studies** | **No. Mesh Studies** |
| --- | --- | --- | --- | --- | --- | --- | --- | --- | --- | --- | --- | --- |
| BESAQ | Body Exposure During Sexual Activity Questionnaire | Generic | Cash et al. (2004). | To assess a person’s self-conscious focus on their body’s appearance during sex and their desire to avoid exposure of their body to sexual partners | 28 | 0 | Likert | Not specified | 1 | 0 | 0 | 0 |
| BIPOP | Body Image in Pelvic Organ Prolapse | Pelvic organ prolapse | Lowder et al. (2014). DOI: 10.1016/j.ajog.2014.03.019 | To assess how prolapse affects a woman's body image | 10 | 0 | Likert | Not specified | 1 | 0 | 0 | 0 |
| BIQLI | Body Image Quality of Life Inventory | Generic | Cash & Fleming (2002). DOI: [10.1002/eat.10033](https://doi.org/10.1002/eat.10033) | To measure the impact of body image on a person’s life | 19 | 0 | Likert | Not specified | 1 | 0 | 0 | 0 |
| BIS | Body Image Scale | Cancer | Hopwood et al. (2001). DOI: [10.1016/s0959-8049(00)00353-1](https://doi.org/10.1016/s0959-8049(00)00353-1) | To assess body image changes in patients with cancer | 10 | 0 | Likert | 1 week | 5 | 2 | 1 | 0 |

* Alternative terms or abbreviations for instrument

** Response categories - Likert: categorical/continuous data; NRS: numerical rating scale, continuous data; Dichotomous: categorical data, Yes/No responses; Nominal: categorical data, 3+ response options; VAS: visual analogue scale, continuous data; Free text: textual data
