# Supplementary material for: Patient‐Reported Outcome Measures Used to Assess Surgical Interventions for Pelvic Organ Prolapse, Stress Urinary Incontinence and Mesh Complications: A Scoping Review for the Development of the APPRAISE PROM
Source: BJOG. 2025 Sep 24;133(2):218–27. doi: 10.1111/1471-0528.18355 (PMC12678042; doi:10.1111/1471-0528.18355)
Supplement: Supplementary file 18 — Table S9: Table of PREMs—extracted data. [file BJO-133-218-s018.docx]

**Table S9: PREMs – Extracted Data**

| **PROM (short title)** | **PROM (long title)** | **Study reporting psychometric properties** | **PROM Aim** | **No. Core items** | **No. Bother items** | **Type of Response Categories**** | **Recall Period** | **No. POP Studies** | **No. SUI Studies** | **No. POP/SUI Combined Studies** | **No. Mesh Studies** |
| --- | --- | --- | --- | --- | --- | --- | --- | --- | --- | --- | --- |
| COPS-D | Core Questionnaire for the Assessment of Patient Satisfaction for General Day care | Kleefstra et al. (2012). DOI: 10.1186/1472-6963-12-125 | To assess patient satisfaction with day care in hospitals | 27 | 0 | Likert | Not specified | 1 | 0 | 0 | 0 |
| CSQ | Client Satisfaction Questionnaire | Attkisson & Zwick (1982). DOI: 10.1016/0149-7189(82)90074-x | To assess consumer satisfaction with health services | 8 | 0 | Likert | Not specified | 0 | 0 | 1 | 0 |
| ICIQ-S | International Consultation on Incontinence Questionnaire - Satisfaction | Uren et al. (2020) DOI: 10.1111/bju.15091 | To assess satisfaction after urological surgery | 14 | 2 | Likert, NRS, free text | Since most recent surgery | 0 | 0 | 0 | 1 |

* Alternative terms or abbreviations for instrument

** Response categories - Likert: categorical/continuous data; NRS: numerical rating scale, continuous data; Dichotomous: categorical data, Yes/No responses; Nominal: categorical data, 3+ response options; VAS: visual analogue scale, continuous data; Free text: textual data
