# Supplementary material for: Patient‐Reported Outcome Measures Used to Assess Surgical Interventions for Pelvic Organ Prolapse, Stress Urinary Incontinence and Mesh Complications: A Scoping Review for the Development of the APPRAISE PROM
Source: BJOG. 2025 Sep 24;133(2):218–27. doi: 10.1111/1471-0528.18355 (PMC12678042; doi:10.1111/1471-0528.18355)
Supplement: Supplementary file 19 — Table S10: Table of outcomes assessed by each instrument. [file BJO-133-218-s015.pdf]

PROM	Applied for POP?	Applied for SUI?	Applied for Mesh?	No. of Domains/Sub-Scales	Culminative No. Studies	Urinary	Bowel	Vaginal	Prolapse	Gastrointestinal (incl. Nausea/Vomiting)	Scarring	Sexual Function	Pain & Discomfort	Sensory (Sight, Hearing, Speech, Touch, Smell)	Emotional Wellbeing/Mental Health	Spirituality/Religion	Social Life	Social Support	Work/ Employment/Finances	Intimate Relationships	Daily Activities (incl. Household Tasks)	Role Functioning	Exercise	Physical Functioning/ Mobility	Fertility, Pregnancy & Birth	Menopause	Travel	Cognitive/ Mental Functioning	Body Image/ Identity	Self-Care (Dressing, Bathing, Grooming, Pad Use)	Sleep	Energy & Vitality	Co-Morbidities	Complications/Side Effects of Treatment	Improvement/Change	General Health/ Overall QoL	Medical Consumption (Costs/Accessibility etc)	Satisfaction with Care	Information and Advice	Aftercare	Autonomy (Decision-Making, Participation)	Satisfaction with outcome	Satisfaction with Healthcare Environment	No. patient-reported outcomes/experiences measured
15D*	Y	Y		1	10							X	X		X						X			X				X		X	X	X				X								10
AAS*	Y	Y		1	6							X									X		X	X																				4
ADL*	Y	Y		1	3	X	X														X			X						X														5
ALDS*		Y		1	1																X		X	X			X			X														5
APFQ*	Y			4	7	X	X	X	X			X	X				X													X	X													9
BAI	Y	Y		1	2										X																													1
BBUSQ-22	Y	Y		4	11	X	X																																					2
BDI	Y	Y		2	3										X																													1
BDI-SF	Y			1	1										X																													1
BESAQ*	Y			1	1							X								X									X															3
BFLUTS*	Y	Y	Y	5	28	X						X								X	X															X								5
BIPOP*	Y			1	1							X			X					X									X			X												5
BIPQ*	Y			1	1										X																				X	X								3
BIQLI*	Y			1	1							X			X		X		X	X			X						X	X														8
BIS	Y	Y		1	8																								X															1
BISF-W*	Y			1	1							X								X																								2
BRIQ	Y			4	1										X																													1
CARE*	Y			4	1					X			X								X			X			X	X																6
CCCS	Y			1	40		X																																					1
CCI	Y	Y		1	3																												X											1
CCIS	Y			1	48		X																																					1
CIRS	Y			1	1																												X											1
CLSS	Y			1	1	X																																						1
COPS-D*	Y			8	1																																	X	X	X	X		X	5
CRADI	Y			1	7		X																																					1
CRADI-8	Y			1	13		X																																					1
CRAIQ*	Y			1	2		X								X		X				X		X	X			X																	7
CRAIQ-7*	Y	Y		1	6		X								X		X				X		X	X			X																	7
CSI*	Y			2	1	X							X		X								X	X				X			X	X	X											9
CSQ	Y	Y		1	1																																	X						1
DIS	Y	Y		1	11	X																																						1
DN4			Y	1	2								X																															1
DRS-PFD*	Y	Y	Y	1	10																																				X	X		2
ePAQ-PF*	Y	Y	Y	5	11	X	X	X	X			X	X				X			X			X	X					X	X	X					X		X				X		16
EPIQ*	Y	Y		5	4	X	X	X	X			X								X					X	X										X								9
EQ-5D-3L	Y	Y		6	8								X		X							X		X						X						X								6

EQ-5D-5L	Y	Y	Y	6	8								X		X							X		X						X						X								6
EQ-VAS	Y		Y	1	4																															X								1
FAS*	Y			1	1																X						X	X		X														4
FIQL*	Y			4	8		X					X			X		X										X		X	X		X				X								9
FISI	Y			4	17		X																																					1
FSDS*	Y	Y		1	6							X			X					X																								3
FSDS-R*	Y			1	2							X			X					X																								3
FSFI*	Y	Y	Y	1	102			X				X	X																															3
GAD-7	Y			1	1										X																													1
GDS-SF	Y			1	1										X																													1
GHQ-12*	Y	Y		1	1										X				X									X	X		X													5
GIQLI*	Y			1	6		X			X			X		X																X	X				X								7
Godin-Shephard	Y			1	1																		X																					1
GRISS	Y	Y		7	3							X																																1
GUTSS*		Y		1	2																														X			X	X			X	X	5
HADS	Y	Y		2	2										X																													1
HUI		Y		7	2								X	X	X									X	X			X		X														7
IADL	Y			1	1																X																							1
ICIQ-B	Y			5	3		X					X																								X								3
ICIQ-FLUTS	Y	Y	Y	1	30	X																																						1
ICIQ-FLUTSsex*		Y		1	1	X						X	X																															3
ICIQ-LUTSqol*	Y	Y		1	8	X						X			X		X		X	X	X	X	X				X		X	X	X	X					X								15
ICIQ-OAB	Y	Y		1	7	X																																						1
ICIQ-S*			Y	1	1																													X	X			X	X	X	X	X		7
ICIQ-UI-SF	Y	Y	Y	1	259	X																																						1
ICIQ-VS*	Y	Y	Y	3	10		X	X	X			X	X							X																								6
ICS/IUGA*	Y	Y	Y	4	9								X																					X										2
IIQ*	Y	Y		1	67	X									X		X				X			X			X																	6
IIQ-7*	Y	Y	Y	1	291	X									X		X				X			X			X																	6
iMCQ	Y			2	2																																X							1
IPAQ		Y		1	3																			X																				1
iPCQ	Y			2	2														X																									1
IPSS	Y	Y	Y	2	25	X																														X								2
I-QOL*	Y	Y	Y	1	69	X						X			X		X												X	X	X													7
ISI	Y	Y	Y	1	52	X																																						1
ISSI		Y	Y	1	3	X																																						1
KESS*	Y			1	6		X			X			X																															3
KHQ*	Y	Y	Y	7	88	X						X			X		X			X	X	X	X	X			X		X	X	X	X					X								15
LCB	Y	Y		1	1																																			X				1
LSS		Y		1	1							X																																1
MESA	Y	Y	Y	2	44	X																																						1
M-ISI*		Y	Y	2	2	X											X				X									X														4
MPQ		Y	Y	3	2								X																															1
MPQ-SF	Y			2	2								X																															1
NPSI	Y	Y		4	1								X	X																														2
OAB-q*	Y			1	2	X									X		X	X		X							X				X	X												8
OAB-q-SF	Y	Y		2	3	X																														X								2
OABSS	Y	Y		1	16	X																																						1
OAB-V8	Y	Y		1	4	X																																						1
ODS	Y			1	18		X																																					1
PAC-QOL*	Y			5	9		X						X		X		X										X			X					X	X								8
PAC-SYM	Y			1	3		X																																					1
PCS	Y	Y	Y	1	2								X																															1
PFBQ*	Y	Y		1	1	X	X	X				X	X																															5

PFDI	Y	Y	Y	3	64	X	X		X																																		3
PFDI-20	Y	Y	Y	3	276	X	X		X																																		3
PFIQ*	Y	Y	Y	3	38	X	X		X						X		X				X		X	X			X																9
PFIQ-7*	Y	Y	Y	3	161	X	X		X						X		X				X		X	X			X																9
PGI-C	Y	Y	Y	1	15																														X									1
PGI-I	Y	Y	Y	1	341																														X									1
PGI-S	Y	Y	Y	1	39																															X								1
PHQ-9	Y	Y		1	9										X																													1
PILL*	Y	Y		1	1		X			X			X	X																	X		X											6
PISQ-12*	Y	Y	Y	1	275	X	X	X	X			X	X		X					X																								8
PISQ-31*	Y	Y		1	15	X	X	X	X			X	X		X					X																								8
PISQ-IR*	Y	Y		1	27	X	X	X	X			X	X		X					X																								8
PONV	Y			1	2					X																																		1
POPDI	Y			1	12				X																																			1
POPDI-6	Y	Y		1	45				X																																			1
POPIQ*	Y			1	2				X						X		X				X		X	X			X																7
POPIQ-7*	Y			1	5				X						X		X				X		X	X			X																7
POP-SS	Y			1	9				X																																			1
POSAS	Y			1	1						X																																	1
PPBC	Y	Y		1	2	X																																						1
PPIUS		Y	Y	1	9	X																																						1
P-QOL*	Y	Y		9	62	X	X	X	X			X	X		X		X	X		X	X	X	X	X			X			X	X	X				X								19
PROMIS Pain Intensity SF	Y			1	1								X																															1
PROMIS Pain Interference	Y			1	1								X																															1
PROMIS-SexFS	Y			11	1							X																																1
PSAQ	Y			5	1						X																																	1
PSR-13*	Y			1	1		X						X						X				X	X							X	X			X	X								9
PSR-15*	Y			1	2		X						X						X				X	X						X	X	X			X	X								10
QoR-15*		Y		2	1					X			X	X	X			X	X											X	X	X								X	X			11
QoR-40*	Y			2	1					X			X	X	X			X	X		X							X		X	X	X				X		X	X					14
QS-F*	Y			4	1	X						X	X	X	X					X				X				X			X	X				X								11
RI-10*	Y			1	2								X								X										X	X			X							X		6
RUIS		Y		1	2	X																																						1
SDS-PFD	Y	Y	Y	1	8																																				X			1
SEAPI-QMM*	Y	Y	Y	1	21	X									X									X												X								4
SF-12*	Y	Y	Y	1	24								X		X		X		X		X	X	X	X								X			X	X								10
SF-36*	Y	Y		1	55								X		X		X		X		X	X	X	X						X		X			X	X								11
SII*		Y		1	2	X						X					X													X														4
SIP*		Y		1	2							X		X	X		X				X	X		X			X	X		X	X	X												12
SPS	Y			1	4								X																															1
SPS-Q*	Y			1	1	X	X	X	X				X																															5
SSAQ	Y			5	2							X																																1
SSI	Y	Y		1	8	X																																						1
SSQ-8*	Y	Y	Y	1	10														X		X		X															X				X		5
St Marks	Y			1	4		X																																					1
STAI		Y		2	1										X																													1
SUIQQ*		Y	Y	4	5	X						X																								X						X		4
SWLS	Y			1	1																															X								1
TBS		Y		1	1	X																																						1
UDI	Y	Y	Y	1	97	X																																						1
UDI-6	Y	Y	Y	1	365	X																																						1
UIOS		Y	Y	1	1	X																																						1
UIQ*	Y	Y		1	4	X									X		X				X		X	X			X																	7
UIQ-7*	Y	Y	Y	1	7	X									X		X				X		X	X			X																	7

UISS	Y	Y		1	14	X																																						1
ULCQ		Y		1	1	X																																						1
UPS	Y	Y		1	9	X																																						1
USP	Y	Y	Y	1	4	X																																						1
USS		Y		1	2	X																																						1
WHOQoL-BREF*	Y			4	2							X	X		X	X	X	X	X		X			X			X	X	X		X	X					X	X						16
Wong Baker Pain Scale	Y	Y		1	8								X																															1
