# Supplementary material for: Patient‐Reported Outcome Measures Used to Assess Surgical Interventions for Pelvic Organ Prolapse, Stress Urinary Incontinence and Mesh Complications: A Scoping Review for the Development of the APPRAISE PROM
Source: BJOG. 2025 Sep 24;133(2):218–27. doi: 10.1111/1471-0528.18355 (PMC12678042; doi:10.1111/1471-0528.18355)
Supplement: Supplementary file 20 — Table S11: Table of non‐English language PROMs. [file BJO-133-218-s004.docx]

**Table S11: Non-English Language PROMs**

| **PROM (short title)** | **PROM (long title)** | **Country of origin** | **PROM aim** | **No. of POP studies** | **No. of SUI studies** | **No. POP/SUI combined studies** | **No. of Mesh Studies** |
| --- | --- | --- | --- | --- | --- | --- | --- |
| Contilife | Contilife | France | To assess the impact of urinary incontinence on quality of life | 1 | 5 | 0 | 1 |
| DDI | Defectation Distress Inventory | Netherlands | To measure defecation symptoms and impact on daily life | 20 | 1 | 1 | 1 |
| Ditrovie | Dirovie | France | To assess urge urinary incontinence | 1 | 4 | 1 | 0 |
| Genital organ prolapse short form | Genital Organ Prolapse Short Form | Sweden | To accurately and reliably identify women with genital organ prolapse | 1 | 0 | 0 | 0 |
| GFPFQ | German Female Pelvic Floor Questionnaire | Germany | To assess pelvic floor function | 8 | 0 | 0 | 0 |
| Herrold Outlet Obstipation Score | Herrold Outlet Obstipation Score | Germany | To assess impact of chronic constipation | 1 | 0 | 0 | 0 |
| Holschneider Score | Holschneider Score | Germany | To measure the severity of faecal incontinence and to evaluate its impact on the quality of life | 1 | 0 | 0 | 0 |
| Ingleman Sundberg Score | Ingleman Sundberg Score | Sweden | To assess the severity of stress urinary incontinence | 1 | 2 | 0 | 0 |
| IOQ | Incontinence Outcome Questionnaire | Germany | To assess quality of life and patient-reported outcome after mid-urethral slings | 0 | 6 | 0 | 2 |
| MHU | Mesure du Handicap Urinaire (Measurement of Urinary Handicap) | France | To measure urinary symptoms | 4 | 5 | 1 | 2 |
| NSF-9 | Nine Questions on Sexual Functioning | Netherlands | To assess sexual function, pain and satisfaction | 0 | 1 | 0 | 0 |
| PPSSQ | Pelvi-Perineal Surgery Sexuality Questionnaire | France | To evaluate sexual health, in a population of sexually active women or not, who have surgery for stress urinary incontinence or pelvic organ prolapse with or without mesh reinforcement | 1 | 0 | 0 | 0 |
| QSD | Questionnaire for Screening Sexual Dysfunctions | Netherlands | To determine the presence, frequency, and experienced discomfort of sexual dysfunctions. | 3 | 0 | 0 | 0 |
